# The use of technology in diabetes in pregnancy: a position statement of expert opinion from the association of medical diabetologists (AMD), the Italian society of diabetology (SID) and the interassociative diabetes and pregnancy study group

**DOI:** 10.1007/s00592-025-02592-2

**Published:** 2025-11-10

**Authors:** Veronica Resi, Cristina Bianchi, Silvia Burlina, Valeria Grancini, Elisa Manicardi, Maria Masulli, Antonietta Maria Scarpitta, Gian Pio Sorice, Raffaella Fresa

**Affiliations:** 1 Endocrinology Unit, Fondazione IRCCS Cà Granda - Ospedale Maggiore Policlinico, Milan, Italy; 2 Section of Diabetes and Metabolic Diseases, Azienda Ospedaliero-Universitaria Pisana, Pisa, Italy; 3 Diabetes and Endocrinology Unit, ULSS8 Berica, Arzignano, Veneto, VI Italy; 4 Diabetes Unit, AUSL Reggio Emilia, Reggio Emilia, Italy; 5 Department of Clinical Medicine and Surgery, Federico II University, Naples, Italy; 6 Unit of Endocrine and Metabolic Diseases “Paolo Borsellino” Hospital, ASP Trapani, 91025 Marsala, Italy; 7 Department of Precision and Regenerative Medicine and Ionian Area, Section of Internal Medicine, Endocrinology, Andrology and Metabolic Diseases, University of Bari Aldo Moro, Bari, Italy; 8 Diabetology Outpatient Clinic, Asl Salerno, District 63, Salerno, Italy

**Keywords:** Pregnancy, Gestational diabetes, Automated insulin delivery, Insulin pump, Type 1 diabetes, Type 2 diabetes, Delivery, Continuous glucose monitoring, Do it yourself artificial pancreas systems

## Abstract

Over the last 10 years, the number of women with diabetes during pregnancy has increased steadily. Maternal glycaemic control is the most important factor influencing maternal and neonatal outcomes, and technological advances have become integral to the evolution of diabetes care during pregnancy. However, rapid technological development must be accompanied by the equally rapid dissemination of information. In particular, knowledge of the availability of automated insulin delivery (AID) systems for managing type 1 diabetes in pregnancy, and of glucose continuous monitoring (CGM) systems for gestational and type 2 diabetes, needs to be increased. The AMD-SID Italian Diabetes and Pregnancy Study Group, supported by the Technology and Diabetes Study Group, has produced this position paper of expert opinion to review the main international guidelines and current evidence on new technologies for the management of pregnancy in women with GDM, type 1 and type 2 diabetes, and to provide detailed suggestions for the use of commercially available systems in clinical practice.

## Introduction

Many efforts have been made to develop and improve the organisation of antenatal services in the diabetes units of the Italian National Health Service to ensure appropriate care for women with pregnancies complicated by diabetes [1]. Nevertheless, adverse maternal and neonatal outcomes are still more common than in pregnancies not complicated by diabetes [2]. Optimal glycaemic control before conception, throughout pregnancy, and in the peripartum period reduces these risks, but is difficult to maintain due to the progressive physiological hormonal changes of pregnancy. These targets are very strict and difficult to achieve, particularly because of the high risk of hypoglycaemia associated with them, the difficulty of managing the progressive increase in insulin resistance as pregnancy progresses, and the abrupt decrease in insulin needs after delivery [3]. While newer studies about systems, which combine CGM with insulin pumps and algorithms to adjust insulin delivery, offer significant potential benefits for managing diabetes during pregnancy show promise, earlier reviews [4], including those from Cochrane [5], noted a lack of robust evidence and conflicting results from limited trials, especially regarding severe neonatal outcomes. Nevertheless, the available technological tools certainly help to achieve recommended metabolic targets by reducing patient burnout. Of course, optimising glycaemic compensation is essential but not sufficient to ensure a safe pregnancy. In particular, during the pregnancy planning phase, it is necessary to pursue certain goals that may influence the pregnancy outcome (Box 1).

**Table:** 

Box 1	
●Encourage the patient to adopt a healthy lifestyle (avoidance of smoking and alcohol, regular physical activity) ●Recommend a diet adequate in calories and carbohydrates, preferably complex carbohydrates, with a low glycaemic index ● Evaluate the most appropriate technology and/or increase knowledge of the technology already in use ● Achieve recommended glycaemic targets ● Screening for chronic complications ● Supplementation with folic acid ● Evaluate and possibly modify drugs not allowed in pregnancy	

**Fig. 1:**
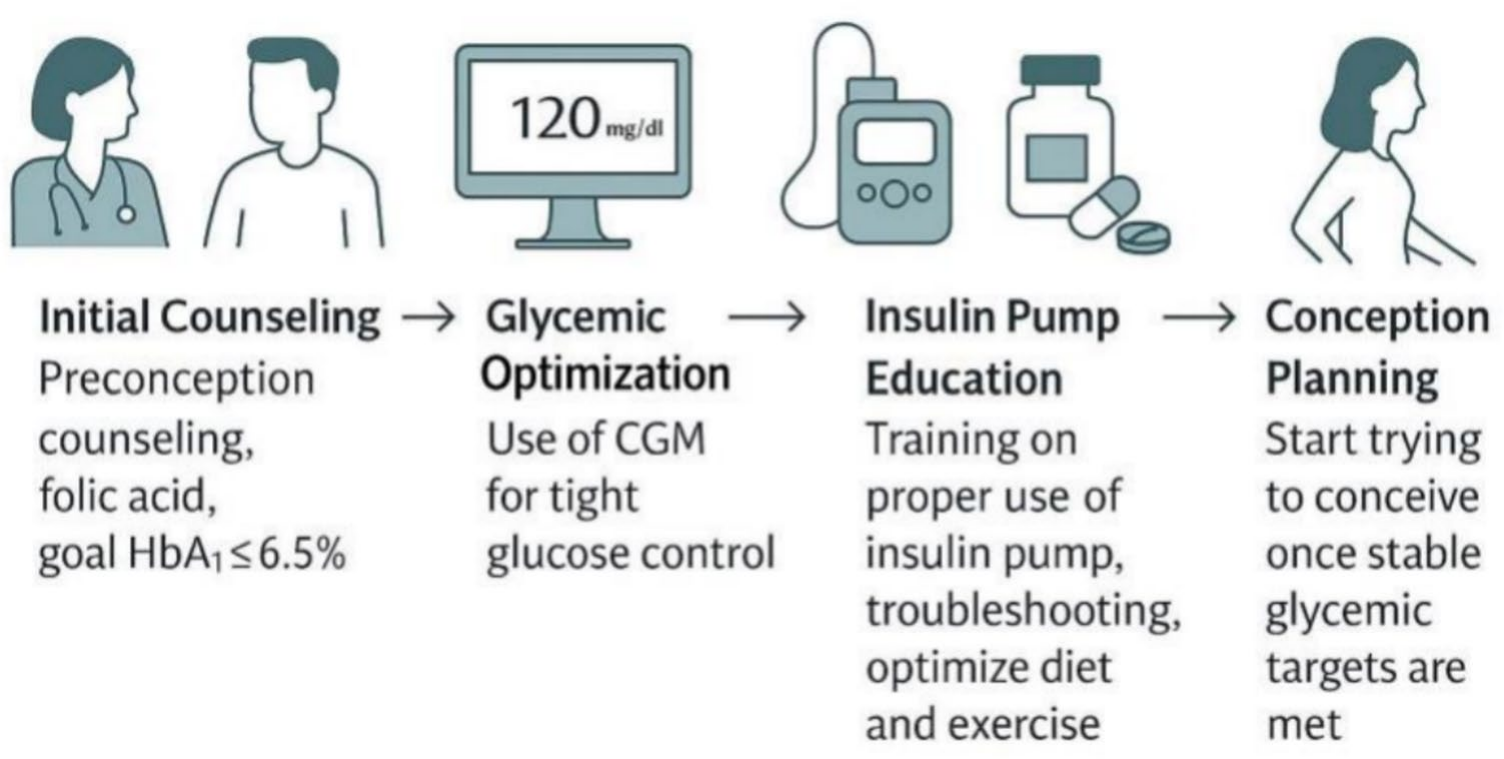
Preconception planning in type 1 diabetes: a technology-assisted roadmap to conception

The use of technology in pregnancy is increasing. Knowledge of new technological systems is essential to address the unique challenges that healthcare professionals and women face in managing diabetes in pregnancy. New research in technology and proven benefits in clinical trials need to be matched by equally rapid dissemination of information. As technology evolves, it is necessary to learn how to safely use increasingly innovative, efficient and advanced systems, and to develop expert and multidisciplinary teams to ensure adequate care for patients with diabetes during pregnancy [1, 6]. For current diabetes technology to be used effectively and safely in pregnancy, it is essential to emphasize that healthcare professionals should operate within a structured multidisciplinary team composed of experienced specialists who possess a comprehensive understanding of both the capabilities and limitations of current technologies in the context of pregnancy: the team should be composed by diabetologists, dietitians/nutritionists, specialized nurses and gynaecologists. It is also important to underline that the use of advanced technologies during pregnancy requires considerable commitment from the patient, who must be adequately motivated and educated in their use, preferably already in the pregnancy planning phase in the case of pre-pregnancy diabetes (Fig. 1).

## Methods and aim

The AMD-SID Diabetes and Pregnancy Study Group, supported by the AMD-SID Technology and Diabetes Study Group, is committed to producing a document based on current scientific evidence and clinical practice on the use of technology in pregnancy, from monitoring systems to the latest evidence on integrated systems in pregnancy complicated by diabetes. A narrative literature review was conducted to support the formulation of this position statement. The authors performed a structured search of PubMed/MEDLINE and Embase database up to July 2025, using combinations of the following keywords: “automated insulin delivery,” “closed-loop insulin,” “type 1 diabetes,” “pregnancy,” “glycaemic control,” “hypoglycaemia,” and “time in range.” Preference was given to randomized controlled trials (RCTs), systematic reviews, real-world evidence (RWE), and clinical guidelines published in English. The selection of evidence was guided by clinical relevance and consensus among the expert authorship group, rather than a formal grading system (e.g., Delphi method or estimate-talk-estimate approach) or systematic review process. Instead, this document reflects expert opinion informed by clinical experience and evidence currently available. The document was subsequently reviewed and endorsed by the National Committees of both Diabetes Italian Associations: Associazione Medici Diabetologi (AMD) and the Società Italiana di Diabetologia (SID). The guidance and suggestions provided herein are intended to support clinical practice and foster awareness and training among healthcare professionals involved in managing pregnancies complicated by diabetes. Given the rapid evolution of this field, the content may be subject to change as new evidence emerges, particularly from large-scale studies.

## Blood glucose monitoring in pregnancy

### Introduction

Due to the physiological increase in red blood cell turnover during pregnancy, glycated hemoglobin (HbA1c) levels decrease [7, 8]. In addition, because HbA1c is an average of glucose levels, it may not adequately reflect postprandial hyperglycemia, which is associated with outcomes such as macrosomia. Therefore, HbA1c is used as a secondary measure of glycemic outcomes in pregnancy [6]. HbA1c target is < 6 –6.5% (42–48 mmol/mol); lower HbA1c—6% (42 mmol/mol) is optimal if it can be achieved without significant hypoglycemia [6, 9]. Blood glucose monitoring is a fundamental tool in pregnancies complicated by pre-existing type 1 or type 2 diabetes (T1D or T2D) or gestational diabetes mellitus (GDM). National and international guidelines recommend structured blood glucose monitoring, with varying intensity depending on the prescribed therapy (dietary management or insulin therapy) [6–11]. Three options are currently available for blood glucose monitoring: capillary blood glucose monitoring (self-monitoring blood glucose, SMBG); continuous glucose monitoring with real-time data available to the patient (rt-CGM); and continuous glucose monitoring with data available only after a scan by the patient (is-CGM). SMBG has been the gold standard for blood glucose monitoring in pregnancy for many years and remains an excellent solution for patients managed on diet alone (Table 1).

**Table 1:** Glycemic targets in pregnancy by national and international guidelines

	SID-AMD [9]	ADA [6]		ACOG [10]	NICE [11]
		T1D or T2D and GDM insulin treated	GDM not insulin treated		
Fasting (mg/dL)	≤ 90	70–95*	< 95	70–95	≤ 95^
1 h post-prandial (mg/dL)	≤ 130	110–140	< 140	110–140	≤ 140
2 h post-prandial (mg/dL)	≤ 120	100–120	< 120	100–120	≤ 115

*Lower glucose limits do not apply to individuals with type 2 diabetes treated with nutrition alone

^^^For women on insulin therapy, the minimum capillary glucose value is 72 mg/dL

However, it is less accepted by patients on insulin therapy, especially basal-bolus regimens, due to the need for multiple capillary blood glucose measurements (up to 8–10 per day). The 2018 Italian Standards for Diabetes Care [9] recommend frequent SMBG for pregnant women:

- For women undergoing dietary management: 75 measurements/month are suggested/recommended.
- For women on insulin therapy: 100–250 measurements/month are suggested/recommended, depending on clinical circumstances.

CGM systems provide continuous measurement of interstitial glucose, with data transmitted to an external unit for storage. These data are available to the patient in real-time (rt-CGM), which can be very useful for both the patient and the clinician in analysing daily glucose trends and making adjustments to ongoing insulin therapy. Intermittent scanned CGM (is-CGM) systems also measure glucose continuously but require the patient to scan the sensor to visualise and save the data. These systems are widely used in clinical practice for insulin-treated patients (T1D or T2D) outside of pregnancy [12]. However, there is growing evidence in the literature to support their use in pregnancies complicated by pre-existing T1D or T2D, or by GDM.

With regard to CGM, achieving strict glycemic targets during pregnancy requires the selection of a CGM device with adequate accuracy. Most rt-CGM devices are approved for use in pregnancy and provide accurate readings [13, 14]. Is-CGM systems are also approved for use in pregnancy, but caution is advised because they report longer times below range (% TBR < 63 mg/dL) compared with rt-CGM, especially overnight, in pregnant women with T1D [15]. To avoid overestimation of hypoglycemia, episodes of hypoglycemia detected by is-CGM should be confirmed with a glucometer. In peri-partum management, the technical guidelines for is-CGM and rt-CGM devices indicate that the use of diathermy during surgical procedures may potentially damage device components. This could affect the accuracy of interstitial glucose measurements or compromise the functionality of alerts and data transmission. However, clinical experience with the use of these systems during cesarean delivery has not reported any adverse events. In AIDAPT e CRISTAL trials no specific adverse events are reported regarding the use of CGM during cesarean section [16, 17]. These data suggest that, despite the cautions in the technical guidelines, the use of is-CGM and rt-CGM during caesarean delivery appears to be safe, provided that adequate clinical monitoring is ensured and technical limitations are carefully considered.

### CGM in gestational diabetes mellitus

#### Rationale for use

The potential advantages of SMBG include ease of use, widespread availability and reimbursement by the National Health Service (SSN). Glycaemic targets are well established, although they vary between guidelines. The main limitation is poor patient adherence due to the need for multiple daily checks. In addition, SMBG only provides a snapshot of glucose levels at the time of measurement, with no indication of trends. CGM offers advantages such as ease of use, ability to detect hyperglycaemia or hypoglycaemia, and insight into glucose variability [18]. However, CGM is limited by higher costs and inconsistent reimbursement across Italian regions and.

#### Scientific evidence and recommendations

Data on the use of CGM in GDM are limited, with few trials and small sample sizes. Two meta-analyses have evaluated the effects of rt-CGM in women with GDM [19, 20]. Six randomised controlled trials (RCT) [21–26] involving 482 participants found that CGM use reduced HbA1c by 0.22% compared with SMBG. However, only one trial showed a significant reduction in HbA1c, specifically in women on insulin therapy, suggesting that the benefits of CGM are primarily seen in those using insulin [20]. Women using CGM had less weight gain (mean difference: − 1.17, 95% CI − 2.15 to − 0.19) and their newborns had lower birth weights (mean difference: − 116.26 g, 95% CI − 224.70 to − 7.81). No differences were observed in other maternal or neonataloutcomes [26]. A recent RCT comparing CGM and SMBG in 154 women with GDM and HbA1c < 6% found similar glycemic control, evaluated by Time in Range (TIR) and HbA1c levels antepartum, between the groups. Women in the CGM group had a higher proportion of adequate weight gain (59.7% vs 40.3%, p = 0.046) and lower neonatal birth weight (3123.79 ± 369.58 g vs 3291.56 ± 386.59 g, p = 0.015). No significant differences were observed in other maternal or fetal outcomes, but costs were higher in the CGM group [15]. A large prospective observational study [27] of 1302 women with GDM showed that CGM-derived metrics such as time in range (psTIR), time above range (psTAR), area under the curve (AUC), mean blood glucose (MBG), and glucose variability were positively associated with adverse fetal outcomes, including large-for-gestational-age (LGA) infants. Higher psTAR was also associated with increased neonatal intensive care admissions. Another recent study [28] showed that CGM-derived glucose patterns in 768 healthy pregnant women could predict the development of GDM (diagnosed by OGTT at 24–28 weeks), suggesting potential diagnostic implications. The MAGIC study, a prospective multicentre observational study not yet published, aims to evaluate physiological glucose patterns in pregnancy, their association with fetal outcomes, and the predictive ability of CGM compared with OGTT in identifying LGA and other adverse outcomes. It was an observational study, included a relatively small sample size, lacked randomization, and used heterogeneous devices. Moreover, the short duration of follow-up and absence of standardized outcome definitions limit the strength and generalizability of its conclusions [29].

#### Suggestions for use in clinical practice

Current evidence is insufficient to recommend CGM routine use for clinical and diagnostic purposes (detecting early gestational diabetes and predicting the development of GDM). However, the use of CGM could be considered for women with GDM on insulin therapy to achieve better glycaemic control and improve certain outcomes. When using CGM in clinical practice it is essential to configure CGM platforms with pregnancy-specific targets for normoglycemia, hyperglycemia, and hypoglycemia. For pregnancy, psTIR is defined as 63–140 mg/dL, psTAR > 140 mg/dL, and psTBR < 63 mg/dL.

#### Achieve glycemic targets

Although specific targets for GDM in pregnancy have not been established, expert consensus [30] suggests the following targets:

- psTIR (63–140 mg/dL): ≥ 90%.
- psTAR (> 140 mg/dL): < 5%.
- psTBR (< 63 mg/dL): < 4%.
- psTBR (< 54 mg/dL): < 1%.

### CGM in pre-gestational diabetes (type 1 and type 2 diabetes)

#### Rationale for use

CGM systems (both rt-CGM and is-CGM) offer a greater ability to detect hyperglycemia and hypoglycemia in pregnant women with pregestational diabetes on insulin therapy. The benefits of CGM use during pregnancy include increased TIR and a reduced risk of adverse neonatal outcomes, such as LGA births, macrosomia, shoulder dystocia, neonatal hypoglycemia, and the need for neonatal intensive care unit admission [31, 32]. These findings apply primarily to pregnant women with pregestational T1D, while data on women with pregestational T2D remain limited. A study comparing CGM data between pregnant patients with T1D and those with T2D showed that the latter spent approximately 33% less time in hyperglycemia and less time in hypoglycemia, despite having a similar frequency of nocturnal hypoglycemia to women with T1D [33]. These findings highlight that CGM can also be a valuable tool for patients and clinicians in managing pregnancy in women with pregestational T2D.

#### Scientific evidence and recommendations

Four RCTs have evaluated the use of CGM compared with SMBG alone in pregnant women with T1D [31, 32, 34, 35]. Two trials evaluated the use of is-CGM in women with T1D and T2D, and one trial included women with GDM and reported conflicting results [32, 34]. The first randomised controlled trial of rt-CGM in pregnancy showed no improvement in HbA1c levels or pregnancy outcomes. However, rt-CGM was used intermittently and only 49 (62%) of the 79 women used rt-CGM as per protocol due to technical problems or alarm fatigue [35]. The largest RCT of CGM use in pregnancy to date, the CONCEPTT (Continuous Glucose Monitoring in Women With Type 1 Diabetes in Pregnancy Trial) [31], showed that the use of rt-CGM in addition to SMBG was associated with lower HbA1c concentrations, higher TIR and lower glycaemic variability compared with SMBG alone. Of particular clinical relevance, there was a significant improvement in neonatal outcomes, with a reduction in LGA neonates, NICU admissions for more than 24 h, and neonatal hypoglycaemia. Strengths of this study include the large sample size (325 women) and stratified randomisation based on insulin therapy (MDI vs. CSII) and baseline metabolic control (< 7.5% vs. ≥ 7.5%). As only women with HbA1c between 6.5% (48 mmol/mol) and 10.0% (86 mmol/mol) were included in the study, it remains unclear whether women with lower HbA1c at the start of pregnancy could also benefit from the use of rt-CGM. The CONCEPTT study also showed that the use of CGMs is cost-effective, leads to fewer NICU admissions and has the potential to improve neonatal outcomes at no additional cost [36]. As a result, some international guidelines (UK and Canada) recommend the use of rt-CGM in all women with T1D during pregnancy. Currently, there are no randomised clinical trials on the effects of is-CGM on glycaemia and maternal outcomes in women with T1D. Among the existing studies, one showed that FreeStyle Libre 1 (FSL1) is safe during pregnancy compared to SMBG, with greater treatment satisfaction and similar accuracy regardless of diabetes type, gestational age or BMI [13]. Another prospective observational study showed that FSL1 provided lower glucose estimates compared to SMBG in pregnancy [37]. In addition, simultaneous monitoring with FSL1 and rt-CGM for seven days in early pregnancy showed that FSL1 detected a clinically relevant higher percentage of TBR compared to rt-CGM [38]. Therefore, asymptomatic nocturnal hypoglycaemia detected by FSL1 should not necessarily lead to insulin dose reduction or carbohydrate correction unless confirmed by capillary blood glucose measurement. A multicentre observational study evaluated the potential improvement in metabolic control and pregnancy outcomes with the addition of FSL1 glucose monitoring to standard SMBG in pregnant women with T1D [39]. The addition of FSL1 to SMBG resulted in lower HbA1c levels in the second trimester, although this improvement was not sustained over time. In addition, neonates born to mothers using FSL1 were more likely to experience neonatal hypoglycaemia (27.4% vs 19.1%), with no significant differences in other neonatal outcomes. These findings could be explained by deteriorating metabolic control (as measured by HbA1c) in late pregnancy, which increases the risk of neonatal hypoglycaemia, or by the lower accuracy of FSL1 in providing accurate glucose measurements, leading to suboptimal metabolic management. Newer versions of FSL (i.e. is-CGM FreeStyle Libre 2 and rt-CGM FreeStyle Libre 3) have improved accuracy (lower MARD values) and optional alarms to warn of impending hypoglycaemia or hyperglycaemia [40]. It is not yet clear whether the frequent detection of low glucose levels during pregnancy also occurs with the newer FSL versions. A Chinese study found a moderate correlation between HbA1c and psTIR during pregnancy, suggesting that a psTIR of at least 78% is required to achieve an HbA1c target below 6.0% [46]. In a US study, the correlation between HbA1c and psTIR was strong in the second and third trimesters, but weaker in the first trimester. In addition, GMI showed a strong correlation with HbA1c in all trimesters [42]. In women with T1D, higher CGM-derived metrics from 10 weeks’ gestation were associated with an increased incidence of LGA infants [41–43]. In addition to poorer glycaemic control as assessed by HbA1c, lower psTIR and higher psTAR were predictive of obstetric and neonatal complications [44–46]. The use of CGM reduces the fear of hypoglycaemia and improves the detection of asymptomatic nocturnal hypoglycaemia [47–49]. CGM may also facilitate follow-up via telemedicine where appropriate, thereby increasing patient engagement in diabetes care, but further scientific evidence is needed [50, 51]. There are currently no studies in the literature that have directly analysed the potential benefits of using CGM in pregnancies complicated byT2D. Only one pilot study evaluated the use of is-CGM in 78 women with T2D during pregnancy and showed that the system was well accepted and considered useful by the women [35], but there are no data on the effect of CGM on maternal and neonatal complications. It is therefore not yet possible to determine whether the use of CGM in pregnancies complicated by T2D improves maternal and fetal outcomes, or to set precise targets for CGM in terms of euglycaemia, hyperglycaemia and hypoglycaemia (psTIR, psTAR, psTBR). The 2021 AACE (American Association of Clinical Endocrinology) guidelines [52] currently recommend the use of CGM in pregnant women with T1D or T2D on multiple insulin injections. In the new Endocrine Society and European Society of Endocrinology guideline [53], for pregnant people with Type 2 diabetes (T2D) the panel suggests using either CGM or SMBG for glucose‐monitoring during pregnancy. The guideline notes that although CGM may offer potential advantages in certain subgroups, there is currently limited direct evidence showing CGM is superior to SMBG in T2DM during pregnancy.Importantly, the guideline does not support simplifying glucose targets to a single 24-h CGM target of < 140 mg/dL (7.8 mmol/L); rather, it recommends maintaining the standard pregnancy glucose targets based on fasting and post-prandial values: fasting < 95 mg/dL (5.3 mmol/L), 1-h post-meal < 140 mg/dL (7.8 mmol/L), and 2-h post-meal < 120 mg/dL (6.7 mmol/L). Other national and international guidelines do not provide specific recommendations for women with T2D in pregnancy (Table 2).

**Table 2:** National and international guidelines on the use of CGM in pregnancy

Guidelines	T1D in pregnancy	T2D in pregnancy and GDM
Canadian Guidelines Expert Committee 2018 [54]	Recommended use of CGM only in women with T1D to improve glycemic control and neonatal outcomes	No indication
Italian Standards of Care AMD SID 2018 [9]	Recommended use of rt-CGM in addition to capillary blood self-monitoring to improve maternal glycemic control and neonatal outcomes	No indication
Best Practice Guide ABCD-DTN-UK 2018 [55], NICE2015/2020[11]	Recommend the use of rt-CGM for all women with T1D and is-CGM for those who do not accept rt-CGM	No indication
American Association of Clinical Endocrinology Clinical Practice Guideline [52]	CGM is recommended for pregnant women with T1D treated with intensive insulin therapy	CGM is recommended for pregnant women with T2D treated with intensive insulin therapy
ADA 2025 [6]	Recommended use of CGM in addition to capillary glucose monitoring to reduce the risk of LGA babies and neonatal hypoglycemia	No indication
Endocrine Society and European Society of Endocrinology Joint Clinical Practice Guideline [53]	No indication	In T2DM, either CGM or SMBG can be used during pregnancy

#### Suggestions for use in clinical practice

In order to properly assess CGM glucose reports, it is essential that appropriate pregnancy-specific thresholds for normoglycaemia, hyperglycaemia and hypoglycaemia are set in the platforms used for each device. It is essential to configure CGM platforms with pregnancy-specific targets for normoglycemia, hyperglycemia, and hypoglycemia. For pregnancy, psTIR is defined as 63–140 mg/dL, psTAR > 140 mg/dL, and psTBR < 63 mg/dL. The International Expert Consensus [30] has defined specific targets to improve glycemic control and maternal–fetal outcomes in T1D and T2D pregnancies (Table 3). In women with T1D and in women with T2D treated with MDI, CGM may be considered both during pregnancy planning and during pregnancy to improve glycaemic control. Although specific targets for T2D in pregnancy have not been established, expert consensus [30] suggests the following targets (Table 3). Evaluation of the trend arrows is critical to adjusting mealtime insulin therapy and making the correct correction. For this assessment, existing recommendations for patients with T1D should be followed [54], as there are no specific guidelines for pregnancy.

**Table 3:** Target values for continuous glucose monitoring [30]

	Time in range (TIR) (63–140 mg/dl)	Time above range (TAR) (> 140 mg/dl)	Time below range (TBR)	
			(< 63 mg/dl)	(< 54 mg/dl)
Type 1 diabetes	> 70% (> 16 h 48 min/day)	< 25% (< 6 h/day)	< 4% (< 1 h/day)	< 1% (15 min/day)
Type 2 diabetes^^^	≥ 90%	< 5%	< 4%	< 1%

^^^For T1D target values are recommended, for T2D target values are suggested

### Connected insulin pens and caps in pregnancy

In recent years, technological innovation has also affected the way conventional insulin therapy is delivered, with the introduction of connected insulin pens and pen caps that connect to smartphones, tablets and PCs via dedicated apps [56]. These devices combine additional technologies such as tracking and dose calculation with the proven accuracy of insulin pens [57]. Connected insulin pens can automatically record and/or store and/or transmit data on the last doses of insulin administered. The connected pens (0.5–1 IU depending on the model) have a dose memory display showing the number of units administered and the time since the last administration. Data is transmitted 'wirelessly' via Bluetooth or NFC (near field connectivity) technology, allowing data to be displayed on the applications [58]. Each device is compatible with different insulins: some systems work with rapid insulin analogues, while others can work with both rapid and basal insulin analogues. With the integration of advanced technologies such as sensor-based glucose monitoring some devices are able to transfer recorded insulin data to the compatible connected app, allowing instant visualisation, weekly summaries and a combined glucose and insulin data diary.

In 2017, the InPen^®^ was the first connected insulin pen to receive FDA clearance with a bolus calculator and insulin on board (IOB) estimation. Connected caps are devices attached to the top or side of a disposable insulin pen. These devices can record insulin administrations by measuring the movement of the pen plunger. This allows the device to record the dose and time of insulin administration. The information can be displayed on the pen display or on an application that, in some caps, allows integration with CGM or CGM data platforms.

#### Rationale for use

The use of connected insulin pen devices in the context of pregnancy complicated by diabetes could become a very useful tool in the future, especially for women with T1D treated with MDI who are planning a pregnancy but are unwilling or unable to switch to a CSII system. Sometimes the perceived complexity and patient or healthcare preference do not allow the use of infusion systems such as CSII or HCL. Pregnant women with diabetes treated with MDIs face many complexities that often lead to inappropriate therapy, with dosing mistakes that can have a negative impact on glycaemic compensation. For example, without access to data on the timing and dose of previous injections, it can be difficult to identify doses at short intervals that may put them at increased risk of subsequent hypoglycaemia [59].

#### Scientific evidence and recommendations

The CONCEPTT study showed that pregnant women with T1D who used MDIs and CGMs were more likely to achieve HbA1c targets and had an increased TIR at 24 weeks gestation compared with women who used pumps and CGMs [31]. Studies in people with diabetes treated with MDI have shown that failure to take just two doses of insulin with meals per week is associated with a 0.4% increase in HbA1c, demonstrating the importance of correct and timely mealtime insulin boluses [60, 61]. The use of connected pens has been associated with a reduction in missed insulin doses in the not-pregnant population [62]. In addition, available studies showed that connected pen systems are associated with increased TIR, a reduction in hypoglycaemia and an improvement in HbA1c [62, 63]. The ADA includes connected insulin pens in the Medical Standards of Care recommendations for insulin therapy as a solution to potentially help patients on injection therapy with dose recall, dose recommendation and dose titration [6]. There is currently no literature data or regulatory guidance on the use of connected pens/cap systems in pregnancy, but case reports with their use in women with T1D in pregnancy provide useful suggestions that may be associated with certain advantages in the management of insulin therapy during pregnancy [64].

#### Suggestions for use in clinical practice

The use of these new devices in the management of women with T1D in pregnancy is challenging. Connected insulin pens may have the potential to achieve the ambitious metrics target in the pre-pregnancy state. In particular, the use of a bolus system calculator, such as the InPen^®^, would be useful to consider in pregnancy planning. Personalised insulin-to-carbohydrate ratios, duration of insulin action, insulin sensitivity factors and pregnancy-specific target glucose ranges are also educational tools that were previously only available to patients using an insulin pump, and with this system can be made available to women treated with MDI. By managing low and high blood glucose patterns using the system's insulin adjustment tools, women can be educated about the relationship between insulin, diet and physical activity during pregnancy. They can help modify behaviour, verify missed doses, and provide insulin dose reminders based on retrospective review of collected data. Women with GDM and T2D are usually not used to managing insulin therapy. When they are prescribed insulin therapy during pregnancy, they often have difficulty understanding how the therapy works and how to manage it. The future use of devices, such as connected caps that record the dose and time of insulin administration and integrate with CGM or data platforms for CGM, could help these women to better understand and manage insulin therapy in a short time period such as pregnancy.

## Continuous subcutaneous insulin injections (CSII) in pregnancy

### Introduction

**Fig. 2:**
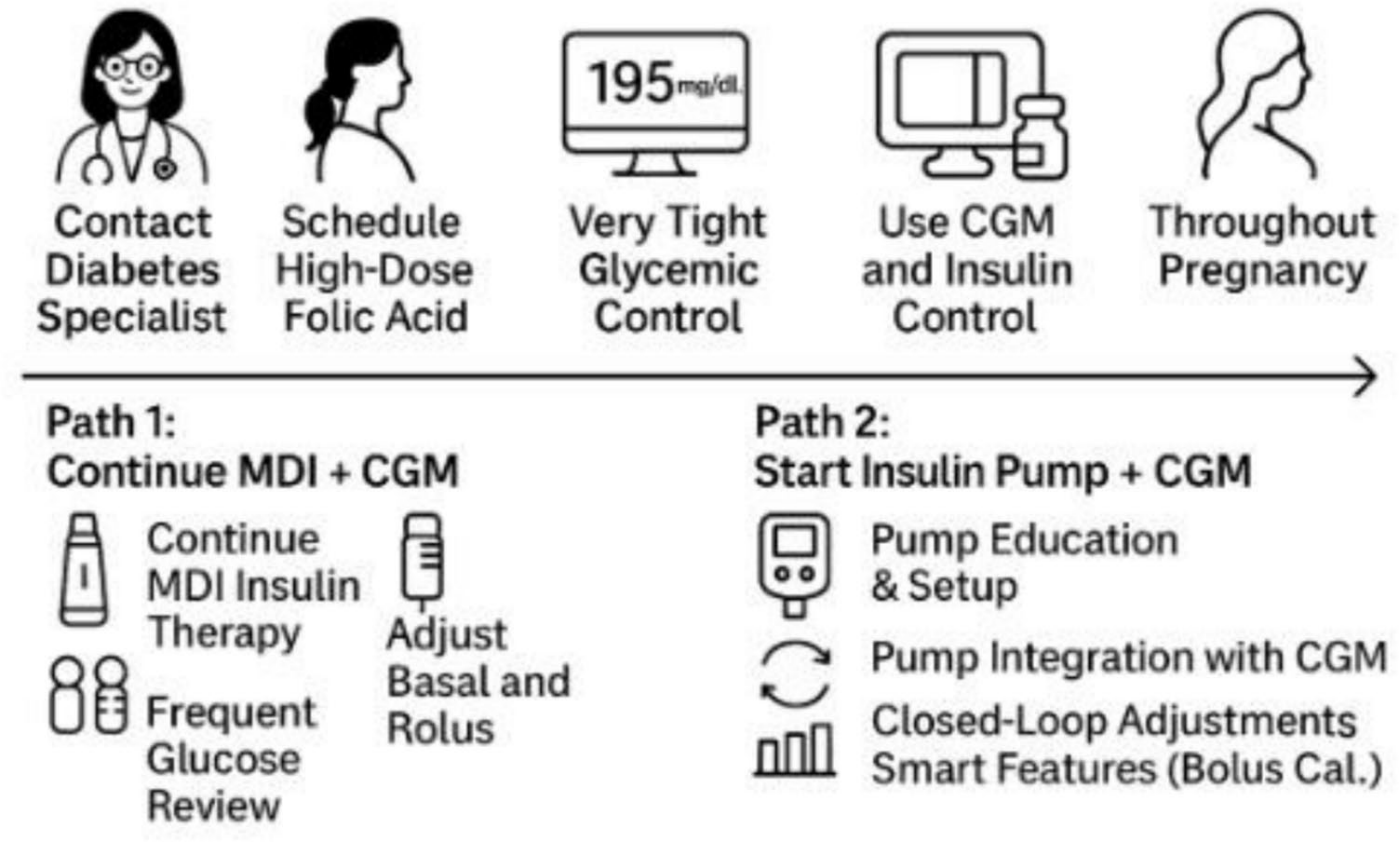
Unplanned pregnancy in type 1 diabetes: two pathways to glycemic optimization. This visual summary outlines a clinical management timeline for women with type 1 diabetes experiencing an unplanned pregnancy. The top sequence highlights urgent early steps: specialist referral, high dose folic acid planning, initiation of tight glycemic control, and CGM integration. Following CGM initiation, two distinct insulin therapy paths are depicted: path1 Continuation of Multiple Daily Injections (MDI) supported by CGM, with structured follow-up and dose titration; path 2 Transition to Insulin Pump Therapy (CSII) with CGM, offering customizable basal profiles, bolus calculators, and optional closed-loop automation (AID). Both approaches require continuous glucose monitoring, education, and multidisciplinary follow-up to reduce pregnancy-related risks and support optimal outcomes

Continuous subcutaneous insulin infusion or insulin pump (CSII) therapy has become increasingly common in the management of diabetes mellitus in pregnancy in recent years. In a 2018 registry study, 74% of pregnant women with T1D were using CSII [64]. This trend towards more frequent use of CSII is likely due to improvements in insulin pump technology and published guidelines that increasingly support the use of pumps in pregnancy. CSII therapy is recommended for women with T1D who are planning to become pregnant. If pregnancy occurs during MDI therapy and targets are not achieved despite optimisation of MDI therapy, CSII therapy is recommended after the end of the embryogenic phase [9–11]. The advantage of CSII therapy is secondary to its ability to more closely mimic physiological insulin secretion, allowing more precise and accurate adjustments in both basal and bolus management. The choice of CSII requires careful patient selection and is a multidisciplinary intervention involving the integration of different levels of care through the use of common and validated protocols dedicated to the education, training and continuity of care of patients using this therapeutic strategy. The choice of CSII system must be discussed with the patient and based on her clinical characteristics and personal history, always taking into account the patient's ability to manage the proposed technology. Different types of CSII systems are currently available, and their specific characteristics make it desirable to prescribe the most appropriate device for each patient from a structural and functional point of view [9]. The choice of the most appropriate and indicated technological therapy should be made at the planning stage of pregnancy and, in particular, the use of most of the AID systems, which are not yet indicated in pregnancy, should be evaluated by the clinician in consultation with the patient [65]. Women considered suitable for CSII therapy must have specific skills: knowledge of carbohydrate counting, use of both the CGM and pump-assist technologies, reliable adherence to medical care; the ability to use the MDI in the event of device malfunction is also required [6]. Women on pump therapy need to be educated in the management of potential device or pump malfunctions, which, if not recognised and managed, may potentially lead to the development of diabetic ketosis/ketoacidosis (DKA) [66] (Fig. 2).

### Stand-alone insulin pumps (CSII) in pregnancy

#### Rationale for use

The potential benefits of using a stand-alone CSII without CGM integration in pregnancy with T1D over traditional MDI therapy are improved glycaemic control (as reflected by glycaemic haemoglobin, HbA1c), reduced glycaemic variability [55], better management of hyperemesis gravidarum episodes, reduced frequency of severe hypoglycaemia, reduced daily insulin requirements and improved quality of life, mainly due to reduced fear of hypoglycaemia and increased lifestyle flexibility [67, 68]. Patients with longer-lasting, more unstable T1D diabetes with unpredictable glycaemic trends may be targeted for the use of CSII over MDI. There is currently no evidence or recommendation supporting the use of stand-alone CSII in pregnant women with type 2 diabetes. Moreover, the available data are limited by small sample sizes, observational designs, and the use of outdated CSII technologies, making their interpretation in the context of current practice difficult.

#### Scientific evidence and recommendations

The available data on the benefits of CSIIs over MDIs in pregnancy are mixed, particularly with regard to the benefits of CSIIs on maternal glycaemic control. Less recent data on CSIIs suggested that their use was associated with an increased risk of hypoglycaemia compared with MDIs [69]. However, more recent data using newer systems appear to support fewer cases of hypoglycaemia in early pregnancy in women using CSII compared with their MDI counterparts [68]. Some studies suggest significantly lower mean HbA1c levels in pregnant women using CSII and report no difference in the risk of hypoglycaemia or diabetic ketoacidosis compared with MDI [70]. However, most of these studies are limited by small sample sizes, observational designs, lack of randomization, and heterogeneity in insulin pump technologies available at the time. A pre-specified analysis of the CONCEPTT study stratified by MDI versus CSII use and HbA1c levels assessed glycaemic control and pregnancy outcomes in women with type 1 diabetes using MDI versus CSII at baseline. With regard to neonatal outcomes, the differences between those using CSII and those using MDI were mixed and not significant [71]. A review of 5 RCTs found no differences in cesarean section rate or perinatal mortality rates [68]; in a large multicentre trial, babies born to mothers using CSII were more likely to be LGA [70]. In a 2018 meta-analysis comparing the two insulin delivery modalities, women treated with CSII compared with MDI reported better glycaemic control in the first trimester, a difference that decreased in subsequent trimesters. CSII therapy was also associated with lower insulin requirements, greater maternal weight gain during pregnancy and an increased risk of neonatal LGA or SGA [72]. These data are consistent with a more recent meta-analysis showing that the group of women treated with CSII had an increased proportion of caesarean sections and LGA newborns [73].

Data on the use of stand-alone CSII systems in pregnancy are not recent and appear to be difficult to interpret. With technological advances in recent years, these considerations may become less relevant: the interpretation of literature data must be analysed taking into account the technology available at the time of the study (Table 4). Overall, recent data suggest that glycaemic control with CSII is more beneficial than with MDI, although there is no clear superiority over MDI therapy in terms of improving maternal and neonatal outcomes. Some considerations regarding the use of CSII alone in pregnancy therefore relate to the use of resources for CSII management: some authors have found that women using CSII require more resources in terms of diabetologist assistance in reviewing patients' glycaemic profiles and modifying insulin regimens during pregnancy [74]. In conclusion, while recent data suggest CSII may improve glycaemic control over MDI, the overall evidence remains inconsistent and difficult to interpret due to methodological limitations and evolving technology. These considerations must be taken into account when extrapolating historical findings to current clinical practice.

**Table 4:** National and international guidelines on insulin pump therapy in pregnancy planning and during pregnancy

Guidelines	Planning	Pregnancy
Canadian Guidelines Expert Committee 2018 [54]	No indication	The use of insulin therapy is recommended both with MDI and pump to achieve glycemic targets (A1C 7.0% or A1C 6.5% if safely achieved)
Italian Standards of Care 2018 [9]	Evaluate CSII therapy if HbA1c > 6.5% despite optimized MDI therapy	The initiation of therapy can only be considered under specific conditions and outside the embryogenesis period CSII therapy should be managed by an expert team for selected women
Best Practice Guide ABCD-DTN-UK 2018 [55], NICE2015/2020 [11]	Evaluate CSII therapy if HbA1c > 6.5% despite optimized MDI therapy	CSII is recommended when glycemic control with optimized MDI is inadequate without disabling hypoglycemia. Initiation of CSII therapy is recommended as soon as it can be safely done
ADA 2025 [6]	No indication	The use of insulin therapy, both with MDI and pump, is recommended to achieve glycemic targets

#### Suggestions for use in clinical practice

When starting CSII therapy during pregnancy, 85 ± 15% of TDD in MDI is recommended: lower requirements should be expected in the case of good compensation (pTIR > 70%) and risk of hypoglycaemia, higher requirements in the case of non-target glycaemic compensation [75]. Frequent contact with the patient is also recommended for rapid optimisation of the pump setting. There is a progressive increase in insulin resistance during pregnancy and therefore the use of CSII requires frequent adjustments to the pump settings, both in terms of basal profile and bolus management (in terms of I:C and FSI). Some references suggest an increase in insulin needs at the end of pregnancy of up to 60% of the TDD, with a possible predominance of bolus needs compared to the basal profile (Table 5). Other possible tools for personalising therapy are the temporary basal profile (TBP), the super bolus and advanced boluses [75]. The TBP allows you to increase or decrease the current basal profile by a certain percentage for a period of time ranging from 15 min to 24 h. The change in basal insulin delivery should be anticipated 1–2 h before the desired change in blood glucose. There are no specific recommendations for use during pregnancy. Indications for a temporary increase in basal rate in pregnancy may include intercurrent illness, corticosteroid prophylaxis for fetal lung maturation, reduced physical activity. Indications for a temporary decrease in basal rate include physical activity, hyperemesis or prolonged nausea (Tables 6, 7).

**Table 5:** THERAPY INITIATION (Before the 20th Week)

CALCULATION OF TOTAL DAILY INSULIN DOSE (TDD) Total Daily Dose (TDD) in MDI × 0.85 ± 15 *Note*: This dose should be reduced in the case of inadvertent hypoglycemia or when the total daily bolus (TDD) > 60%, and should be increased if HbA1c > 64 mmol/mol	
BASELINE PROFILE (Basal rate = Total basal/24)	Method 1: TDD × 0.5 Method 2: Units/kg body weight (according to trimester—Table 6) Method 3: Average value between Method 1 and Method 2
Basal Profile Options:	Option 1: Flat basal rate (PROFILE BASE/24) Option 2: Variable by time slot according to the schedule Option 3: Variable by time slot based on the analysis of glycemic profiles
INSULIN/CARBOHYDRATE RATIO (I/CHO ratio)	Method 1: Use the pre-pregnancy ratio if it works Method 2: Breakfast 300/TDD, Other meals 400/TDD Method 3: Fixed bolus for meals: TDD*0.5/3 if meals are fixed and no carbohydrate counting is done Method 4: Extrapolation from the food diary analysis
SENSITIVITY FACTOR (FSI) Recommended target:	Method 1: Use the pre-pregnancy FSI if it works Method 2: 1700/TDD 90 mg/dl
Post-prandial and Post-correction Delta:	Accept a post-prandial delta up to 60 mg/dl Accept a post-correction delta within 30 mg/dL of the target range after two hours
Adjustment Frequency:	Adjust doses every 2–3 days at the start of therapy, especially for the basal profile during pregnancy
ADJUSTMENT OF THE PROGRAMMING	
CHECK BASELINE PROFILE: TDD CSII × 0.5/24 h. Insulin action time: 2–3 h	
CHECK BOLUSES: adjust by 10–20% at a time based on deviation from the target. Gradually reduce to 4 × during pregnancy	
CHECK CORRECTIONS: adjust by 10–20% if the blood glucose two hours after correction is not within the goal range

**Table 6:** Recommended therapeutic adjustments during pregnancy

State	Pre-pregnancy	1st trimester	2nd trimester	3rd trimester	Full-term pregnancy
Recommended insulin requirement (UI/kg/day)	0.6	0.70	0.80	0.90	1.00
Basal/bolus ratio	50:50				
Basal/bolus ratio (*)	From 50:50 to 35:60			From 35:65 to 25:75	
I/CHO ratio	Parameters ongoing	Breakfast: 300/TDD Lunch & Dinner: 400/TDD		Breakfast: 200/TDD Lunch & Dinner: 300/TDD	
FSI (sensitivity factor)	Parameters ongoing	130/TDD		100/TDD	
Bolus timing before meal	Parameters ongoing	15 min before	20 min before	30 min before	

*Option proposed by the Italian Diabetes and Pregnancy Study Group

**Table 7:** Changes in basal profile per time slot [Basal rate = Basal profile/24 h]

Time slot of the day	ABCD DTN-UK [55]	Guidelines for optimal bolus calculation [75]
Bedtime to up to 3 h after waking	80–100%	0.5 × Basal/24
From 3 h before waking to wake-up	100–120%	1.5 × Basal/24
From wake-up to lunch	80–100%	1.0 × Basal/24
From lunch to dinner	80–100%	1.0 × Basal/24
From dinner to bedtime	100–120%	1.0 × Basal/24

### Sensor-augmented insulin pump with low-glucose suspend (LGS) and predictive low-glucose suspend (PLGS) in pregnancy

#### Rationale for use

Sensor-Augmented Pump Therapy (SAP) consists of a semi-integrated system (open-loop systems) that combines two technologies: a CSII system and a continuous glucose monitoring (rt-CGM) system [76]. These systems allow the insulin therapy to be modified based on blood glucose readings from the sensor. Technological development has allowed an implementation with the introduction of automatic decision algorithms: the Low Glucose Suspend (LGS) function, the pump automatically suspending basal insulin delivery when it detects a glucose level below a set threshold, and the Predictive Low Suspending Glucose Pump (PLSG), which suspends insulin delivery in anticipation of hypoglycaemia. These systems have been shown to reduce HbA1c, to improve TIR without increasing hypoglycaemia and with no increase in the risk of diabetic ketoacidosis (DKA) in T1D patients compared with standard treatment [68]. There are no specific recommendations for the use of SAP in pregnancy: the available evidence and rapid technological evolution have led to some guidelines and recommendations referring to the use of CSII. However, to date, there are no indications or restrictions for the use of these systems in pregnancy. This reflects both the absence of dedicated clinical trials and the fact that insulin use in T2D is often temporary and initiated later in pregnancy, limiting the feasibility and potential benefits of SAP. There is currently no evidence supporting the use of SAP therapy in pregnancy, with available studies restricted to T1D and further limited by small cohorts, observational designs, and outdated technologies.

#### Scientific evidence and recommendations

In a recent prospective observational study, women in pregnancy with T1D treated with SAP/LGS showed better glycaemic control in the third trimester compared with women treated with CSII without rtCGM, but no differences in outcomes such as macrosomia or preterm birth [77, 78]. There is some evidence of a reduced risk of hypoglycaemia with SAP treatment with PLGS in women with T1D during pregnancy: in a recent retrospective Italian study comparing the use of SAP + PLGS with therapy with MDI with rt-CGM, it was found that neither group achieved psTAR and psTIR targets during the first and second trimesters, and that those on MDI with rt-CGM had significantly higher psTIR and lowerTAR; however, TBR (level 1 and 2) values were significantly lower in the SAP + PLGS group in all trimesters [79]. There are no RCTs comparing obstetric and neonatal outcomes in pregnant women with T1D treated with SAP versus CSII-stand-alone SMBG (CSII/SMBG). A retrospective cohort study aimed to evaluate the efficacy of SAP therapy in improving obstetric and neonatal outcomes in pregnant women with T1D compared with CSII/SMBG [80]: this study suggested that SAP appeared to contribute to a reduction in the incidence of LGA in pregnancies complicated by T1D compared with CSII/SMBG therapy. Interestingly, the incidence of LGA in pregnancies complicated by T1D in which SAP was started after conception did not differ from that in which SAP was used before conception. Previous studies have compared obstetric and neonatal outcomes in pregnant women with T1D between CGM users and non-CGM users [67] or between insulin pump users and non-insulin pump users [69, 77].However, the available evidence is limited by small sample sizes, predominantly observational and retrospective designs, lack of randomization, and heterogeneity in device technologies, which restricts the strength and generalizability of these findings.

#### Suggestions for use in clinical practice

The LGS feature stops insulin delivery when the sensor glucose reaches or falls below the set low glucose threshold. Insulin suspension should not be used to treat hypoglycaemia as it does not act quickly enough. In pregnancy, most hypoglycaemic episodes occur 1.5–4 h after the meal bolus in the presence of insulin, due to the large pre-meal boluses required to limit the post-meal glucose spike. After insulin withdrawal, there is a risk of rebound hyperglycaemia, which is particularly important in pregnancy. Rebound hyperglycaemia is even more likely if the person eats fast-acting carbohydrates and basal remains suspended (sometimes called 'double treatment' of hypoglycaemia). To avoid this 'double treatment', if the woman is treating/preventing hypoglycaemia with fast-acting carbohydrates, she must reactivate the basal as soon as euglycaemia is reached. If suspensions always occur at the same time of day or night, the insulin settings (both basal and bolus and correction settings) need to be changed. To date, two systems with a predictive hypoglycaemia (PLGS) function are commercially available. Basal-IQ predicts what the glucose levels will be over the next 30 min and suspends insulin if the sensor glucose is expected to reach or fall below 80 mg/dL, or if the actual sensor glucose is ≤ 70 mg/dL. The Smart Guard system uses the user-selected low level as data and then suspends in relation to it. The PLGS function is activated if the sensor glucose is expected to reach or fall below 20 mg/dL above the low glucose threshold within 30 min and the sensor glucose is ≤ 70 mg/dL above the low glucose threshold. After a delay of at least 30 min, basal insulin delivery is automatically resumed if sensor glucose is at least 20 mg/dL above the low glucose threshold and blood glucose is expected to be 40 mg/dL above the low glucose threshold within 30 min [55]. Therefore, in the case of severe rebound hyperglycaemia, always check whether the woman is 'double-treating', and if the predictive withdrawal function is active, the predictive warning (which usually prompts the person to consume fast-acting carbohydrates) must be switched off, and vice versa. The main difference between the two systems is the flexibility offered by SmartGuard, which allows, for example, a higher lower limit at night and variation during the day, whereas Basal-IQ is restricted to a single set of limits; however, the insulin restart criteria of the BASAL-IQ system are more flexible, potentially reducing the likelihood of rebound hyperglycaemia.

### Automated insulin delivery systems (AID) in pregnancy

**Fig. 3:**
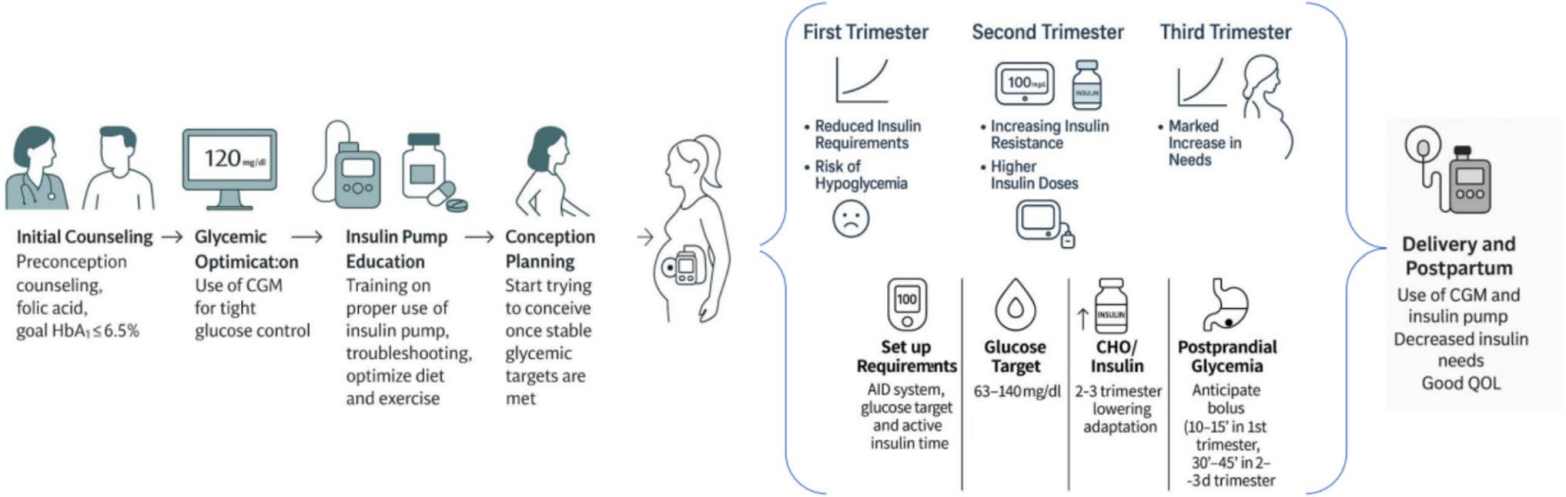
Comprehensive management of type 1 diabetes in pregnancy using advanced diabetes technologies

Achieving and maintaining as near euglycemia as safely possible prior to conception is highly effective in reducing the risk of congenital malformations and decreasing the risk of preterm delivery and admission to neonatal intensive care units according to the ADA Standard of Care [6]; the National Institute for Health and Care Excellence (NICE) guideline [11], recommends achieving an HbA1c level of < 6.5% and maintaining glucose levels within a target range of 70–130 mg/dL. The use of automated insulin delivery (AID) systems is expected to increase the likelihood of safely attaining these tighter glycemic targets by minimizing glucose variability and providing proactive adjustments to insulin delivery. The improvement in metabolic control achieved with AID systems [81, 82] compared with standard care has led to increased use of closed-loop devices in people with T1D, including women of childbearing age. Most current AID systems are not widely recommended during pregnancy mainly because they were not designed or tested for the unique demands of pregnancy, particularly the need for tighter glycaemic control [30] and the dynamic insulin sensitivity that occurs. Healthcare professionals increasingly encounter a clinical dilemma during pregnancy: whether to continue using AID systems despite the lack of formal indication for their use during pregnancy, or to transition patients to conventional pump/MDI approaches aligned with established guidelines [6, 11, 16, 69]. This decision is further complicated by the fact that many women with T1D may be reluctant to discontinue AID systems, particularly if they have experienced improved glycaemic control and quality of life with these technologies prior to pregnancy [83, 84]. CamAPS FX (Dana Diabecare RS and Dana‑i insulin pumps) received CE marking in Europe and the UK for use in people with T1D, including pregnant individuals [85]. In May 2024, the U.S. Food and Drug Administration (FDA) explicitly authorized its use during pregnancy [86]. It’s the first AID algorithm formally approved for pregnancy use by both EU and US regulatory bodies, preceding the CE mark expansion granted to MiniMed 780G in July 2025 [87]. Nonetheless, regulatory approvals vary across regions, and the use of AID systems during pregnancy still requires individualized clinical assessment and close monitoring. The selection of an appropriate insulin delivery system should ideally occur during the preconception planning phase in collaboration with a multidisciplinary team experienced in diabetes and pregnancy care to ensure compatibility with the specific glycaemic targets required in pregnancy [11, 31]. If an automated insulin delivery (AID) system not formally approved for use in pregnancy is selected, its continued use should be considered off-label and requires informed consent. Women must be adequately counselled about the benefits, limitations, and potential risks, and closely monitored by an experienced multidisciplinary team specializing in diabetes and pregnancy [88] (Fig. 3). Despite the rising global burden of T2D in adolescents and women of childbearing age [89], there is currently no indication for the use of AID systems in pregnancy complicated by T2D. Unlike T1D, evidence in T2D is extremely limited, with no randomized controlled trials or robust real-world studies demonstrating clinical or cost-effectiveness [2]. Barriers such as late diagnosis, temporary need for insulin, limited time for patient and provider training, socioeconomic factors, and device accessibility further restrict feasibility [2, 90]. Consequently, AID use in T2D pregnancy remains unsupported by evidence or guidelines, and recommendations cannot currently be made [53].

#### Rationale for use

The rationale for using automated insulin delivery (AID) systems during pregnancy is largely extrapolated from their well-documented ability to reduce glucose variability in non-pregnant individuals with type 1 diabetes [91] without increasing the risk of hypoglycaemia—an important benefit in the context of pregnancy, where tight glycaemic control is essential [92]. In addition, emerging evidence suggests that their use during pregnancy contributes to improved quality of life, including reduced fear of nocturnal hypoglycaemia, decreased daily diabetes management burden, and a heightened sense of normality and autonomy [92].

#### Scientific evidences from randomized controlled trials (RCTs) and real-world evidence (RWE)

In recent years, numerous scientific studies have demonstrated the efficacy and safety of both AID systems formally approved for use during pregnancy and those used off-label in this context. The most consistent studies on the use of AID systems in pregnancy have been conducted with the Cambridge CamAPS FX algorithm. The initial version of the algorithm was tested in the CLIP 1–4 studies [92, 93] while the modified version with adaptable targets was tested in the AiDAPT study [16]. The CLIP 1–2 studies evaluated the safety and efficacy of the algorithm in an inpatient setting overnight (CLIP 1) and for 24 h (CLIP 2). These initial experiences were followed by the randomised cross-over CLIP 3 study, which evaluated overnight hybrid closed-loop (HCL) therapy (with an algorithm target range of 97–124 mg/dL [5.4–6.9 mmol/L]) in a home setting [92]. The study aimed to compare this therapy with standard sensor-augmented pump therapy (SAPT), with the primary endpoint being overnight pregnancy-specific time in range (psTIR). Results showed a significant improvement in psTIR (74.7% vs. 59.5%, P = 0.002), along with lower mean overnight glucose (6.6 mmol/L vs. 7.4 mmol/L, 119 vs 133 mg/dL, P = 0.009), and no increased psTBR. The open-label randomized cross-over CLIP-4 study [84] evaluated the use of AID vs SAPT in 16 T1D women during pregnancy in a home setting day and night for 28 days, separated by a wash-out period (target range used: 104.4–131.4 mg/dL (5.8–7.3 mmol/L); while both interventions achieved a similar pregnancy-specific time in range (psTIR) of approximately 60%, the AID system was associated with fewer hypoglycaemic episodes. The AiDAPT-RCT study [16] enrolled 124 women with T1D by the 16th week of gestation and randomized them to either standard therapy (multiple MDI) or continuous subcutaneous insulin infusion (CSII) with continuous glucose monitoring (CGM) or automated insulin delivery (AID) using the CamAPS FX closed-loop system (DANA insulin pump, Dexcom G6 CGM, CamAPS FX app on Samsung or other compatible smartphones). The closed-loop group employed gestational age-specific glucose targets: 100 mg/dL during the first trimester and 81–90 mg/dL from the second trimester until delivery. The treatment algorithm aimed to achieve a psTIR of 70%, with an intended mean glucose range of 108–117 mg/dL. The intervention group demonstrated a statistically significant improvement in glycaemic control compared to standard therapy. Specifically, psTIR was 68% in the AID group versus 55% in the control group (p < 0.001). Overnight psTIR was also higher (70.8% vs. 56.7%), and mean nocturnal glucose was lower (125 mg/dL vs. 135 mg/dL) in the AID arm. Despite these improvements, the study’s primary endpoint was not achieved, and nocturnal glycaemic control remained suboptimal, as indicated by a psTIR of 70.8% and mean nocturnal glucose of 125 mg/dL, despite the stringent glycaemic targets embedded in the Cambridge algorithm. Limitations identified by the authors included the absence of data on the use of the system’s Boost and Ease-off features, as well as the timing of participant enrollment, with the majority initiating therapy after the first trimester. Notably, women who commenced closed-loop therapy in the first trimester exhibited a 5% higher psTIR by 12 weeks’ gestation compared to those receiving standard therapy—an improvement known to be associated with better obstetric and neonatal outcomes [16]. The CRISTAL study [17] was an open-label, randomized controlled trial involving 92 pregnant T1D women, aimed at assessing the efficacy of the MiniMed^™^ 780G hybrid closed-loop system, which incorporates the SmartGuard^™^ algorithm, in comparison to standard of care (SoC) therapy with the Guardian^™^ Sensor 3 CGM. The primary objective was to achieve a pregnancy-specific time in range (psTIR; 63–140 mg/dL) greater than 70%, utilizing the system’s most intensive settings (glucose target of 100 mg/dL and AIT of 2 h). Although the primary endpoint was not achieved, the closed-loop group demonstrated a modest, non-significant increase in overall psTIR (66.5%) compared to the SoC group (63.2%). However, several secondary outcomes showed statistically significant benefits in favor of the AID system. Nocturnal psTIR was higher in the closed-loop group (75% vs. 67%; *p* = 0.0026), and both overall and nocturnal time below range (psTBR) were significantly reduced (2.5% vs. 4.1%, *p* = 0.0020, and 1.9% vs. 4.2%, *p* = 0.0005, respectively). Furthermore, participants in the closed-loop arm reported higher treatment satisfaction and a reduction in unrecognized hypoglycaemia, with no adverse safety events observed. Despite the inability to meet the primary endpoint, the closed-loop system achieved notably improved nocturnal glycaemic performance, particularly in psTIR and psTBR metrics, even when operating with a glucose target no lower than 100 mg/dL. These findings support the utility of hybrid closed-loop technology in enhancing overnight glycaemic control and patient experience during pregnancy [17]. A prospective real-world study from Spain involving 124 pregnant women with T1D, of whom 59 were using hybrid closed-loop (HCL) systems, reported greater maternal weight gain and higher neonatal birth weight in the HCL group compared to those managed with multiple daily injections (MDI) and continuous glucose monitoring (CGM). No significant differences were observed between the groups in other maternal or fetal outcomes [94]. A recent meta-analysis by Stamati et al. [95], including 13 studies and 450 pregnant women with T1D, assessed the efficacy and safety of automated insulin delivery (AID) systems compared to standard of care (SoC). The analysis demonstrated a significant improvement in psTIR, along with reductions in TAR > 140 mg/dL and > 180 mg/dL, as well as decreased glycaemic variability in the AID group relative to SoC. Furthermore, a recent review by Sobhani et al. [4] compiled data from 24 observational studies involving 243 pregnancies—227 using commercial AID systems and 16 using open-source platforms. The review reported reassuring maternal–fetal outcomes, with a mean psTIR of 71.8% in the third trimester across the cohort. In addition to existing published evidence, the CIRCUIT study [96]—a multicentre, randomized controlled trial investigating the efficacy and safety of automated insulin delivery (AID) in pregnant women with type 1 diabetes—was recently completed and is currently in the final stages of data analysis. Preliminary data from the ADA Scientific Sessions (LB: Pregnancy—Clinical/Epidemiology, June 20, 2025) showed that initiating hybrid closed-loop therapy (t:slim X2 Control-IQ with Dexcom G6) by ≤ 16 weeks’ gestation increased time in the pregnancy glucose range (63–140 mg/dL) by 12.6 percentage points (~ 3 h/day) compared with standard care, with lower mean glucose and reduced time above and below range. Safety outcomes were similar, with no excess severe hypoglycemia or diabetic ketoacidosis [97]. Although full results are pending publication, the study is expected to contribute important evidence to the growing body of literature on the use of closed-loop insulin delivery systems during pregnancy.

#### Evidences from observational studies

In addition to the aforementioned studies, several observational investigations involving small cohorts have contributed valuable real-world evidence on the use of automated insulin delivery (AID) systems during pregnancy. These early studies have played an important role in informing the design of larger randomized trials. One observational study conducted in the United States [98] evaluated a pregnancy-specific closed-loop algorithm (target range 80–100 mg/dL at night and 80–110 mg/dL during the day) using the iAPS platform in combination with Tandem insulin pumps and Dexcom G6 CGM in 10 women with type 1 diabetes. The study documented a 14% absolute increase in psTIR, reaching 76.6%, with an overnight psTIR of 84.8% and a postprandial (2-h) psTIR of 73.4%. Another report described four women using Control-IQ technology in Sleep Mode during early pregnancy. All achieved a psTIR > 70% and a pregnancy-specific time below range (psTBR) < 1.9% by the end of gestation. Furthermore, three of the four participants maintained glucose levels within the target range of 63–140 mg/dL during labour and delivery [99]. Additional case series and reports have described the use of the MiniMed^™^ 670G [100] and 780G systems in pregnancy [101]. Across these reports, consistent improvements in psTIR have been observed, with overnight psTIR exceeding 70% following initiation of closed-loop therapy [101]—particularly when used in the preconception period [102]. Of particular note, a case series involving six women using the MiniMed^™^ 780G system [102] providing data on the use of the SmartGuard^™^ algorithm during both the final two weeks of preconception and the first trimester. In this cohort, the reported TIR (70–180 mg/dL) reached 90%, with a psTIR of 75% during early pregnancy. To date, there are no published clinical trials evaluating the use of the Smart Adjust AID System (Omnipod^®^ 5) during pregnancy. A recent review by Petrovski et al. explicitly states that no data are currently available on its use in this population [103]. A small observational report included in a narrative review by Sobhani et al. describes two pregnant women using Smart Adjust AID, reporting time in range (TIR, 70 to 140 mg/dL) of approximately 68% and 82%, respectively [4]. However, these findings are anecdotal and based on limited sample size, warranting cautious interpretation. Furthermore, a prospective clinical trial evaluating the use of the Smart Adjust AID System (Omnipod^®^ 5) during pregnancy has been registered but results have not yet been published [104]. Although pivotal trials in non-pregnant adults and pediatric populations have demonstrated significant improvements in glycaemic outcomes using the Omnipod^®^ 5 system [105], the lack of pregnancy-specific data limits current clinical application.

#### National and international recommendations

National and international guidelines (Table 8) remain cautious in recommending the routine use of AID systems during pregnancy due to several critical considerations. These include limited access in many healthcare systems, especially where public funding does not cover advanced diabetes technologies, and a range of clinical and technical limitations. Among the key concerns is the necessity for management by a multidisciplinary team with in-depth expertise in the operation of various AID algorithms and their unique features. Importantly, no currently available algorithms—including those with CE and FDA Mark approval—are specifically adapted to accommodate the progressive insulin resistance or delayed insulin absorption characteristic of pregnancy, nor the variability in gastric emptying, which is often patient-specific [2]. Additionally, prolonged basal insulin suspensions by some systems may increase the risk of ketosis or induce post-suspension hyperglycaemic rebounds. Alarm fatigue and concerns about sensor accuracy may also contribute to user burnout, although the performance of newer continuous glucose monitoring (CGM) systems has mitigated some of these issues [30]. Critically, there remains a lack of consistent, high-quality evidence demonstrating clear improvements in maternal or fetal outcomes with the use of AID during pregnancy. Nonetheless, recent updates in guidance reflect a shift toward more permissive recommendations. In December 2023, the National Institute for Health and Care Excellence (NICE) [11] revised its position, endorsing hybrid closed-loop (HCL) systems as a viable option during both pregnancy and preconception, independent of prior criteria such as HbA1c > 6.5%, TIR (70–180 mg/dL) < 70%, or the presence of disabling hypoglycaemia. Similarly, the 2025 ADA Standards of Care now include an updated section on AID use in pregnant individuals with type 1 diabetes, distinguishing between systems with pregnancy-specific targets and those without [6]. The recent 2025 Endocrine Society and European Society of Endocrinology guideline [53],developed using the GRADE approach, suggests the use of a HCL insulin pump in preference to an insulin pump plus CGM without algorithm or MDI plus CGM. This recommendation acknowledges that not all HCL algorithms are validated for pregnancy, and device choice should therefore consider the availability of safety and outcome data in pregnant women with T1D. Furthermore, the guideline emphasizes that insulin adjustment in pregnancy, even when using CGM or HCL, should continue to be guided by fasting and post-prandial glucose targets rather than a simplified 24-h mean glucose value [53].

**Table 8:** National and international guidelines on the use of AID system in pregnancy

Guidelines	Use in pregnancy	Additional notes
Canadian Guidelines 2018 [54]	It remains to be demonstrated whether closed-loop systems can provide benefits when used during pregnancy	No clear conclusion yet
Standard Italiani SID-AMD 2018 [9]	A small RCT on pregnant women with T1D shows clear superiority of the closed-loop system in terms of feasibility, safety, and efficacy compared to SAP	Suggests further study and potential benefits
AACE Guidelines 2021 [52]	The use of AID is not indicated in pregnancy due to higher glucose targets (120–150 mg/dL), which are greater than what is required during pregnancy	Caution regarding higher target glucose levels
NICE 2023 [11]	No restrictions on the use of HCL in T1D during pregnancy and pre-pregnancy planning	Does not support routine use in pregnancy
ADA 2025 [6]	AID systems with specific pregnancy targets are recommended for pregnant individuals with Type 1 diabetes. AID systems without specific glucose targets or algorithms for pregnancy may be considered if used with assisted techniques and monitored by expert care teams	Can be considered for carefully selected individuals with expert guidance
Endocrine Society and European Society of Endocrinology Joint Clinical Practice Guideline [53]	HCL system (insulin pump + CGM + algorithm) can be used in preference to conventional pump + CGM or multiple daily injections + CGM	This is conditional, with low-to-moderate quality evidence, and it cautions that not all algorithms are validated for pregnancy

#### Suggestions for use in clinical practice

The use of AID systems during pregnancy has been shown to support stable nocturnal glycaemic control with a low risk of hypoglycaemia. However, these systems remain less effective in managing daytime glycaemic variability, particularly postprandial hyperglycaemia, which continues to represent a significant clinical challenge [16, 17]. All currently available commercial AID algorithms have specific operational strategies designed to compensate for their limitations in achieving and maintaining recommended glycaemic targets. These strategies—though frequently used in clinical practice—are largely based on expert consensus and clinical experience, rather than on robust clinical trial data [88] (Table 9).

**Table 9:** Integrated overview of clinical settings, management strategies, and adaptations of AID systems in pregnancy

	Set up requirements	Glucose target	Active insulin time (AIT)	CHO/INS	Insulin sensitivity factor (ISF)^§^	Postprandial glycemia		Hypoglycemic trend
						Settings	Strategies	
CamAPS Fx^	Glucose target Body weight TDDI CHO/INS	99 mg/dl up to the 16th week 81 mg/dL overnight 90 during the day from the 16th week Adjustable	Adaptive learning Automatically adjusted	↑ 1st trimester ↓ 2nd and 3rd trimesters	–	Boost function (+ 35% algorithm-driven insulin delivery) if post-prandial blood glucose > 140 mg/dL after 1 h Evaluate correction bolus through the CHO/INS if blood glucose > 180 mg/dl	Use minimum glucose threshold and evaluate if not to enter glucose value to deliver bolus without reverse correction Anticipate bolus 10–15′ in 1st trimester, 30′–45′ in 2nd–3rd	Ease-off mode (increases glucose target by + 45 mg/dL)
Smart Guard^	Glucose target AIT CHO/INS	100 mg/dl (also available from 110–120) Adjustable	2 h Adjustable	↑ 1st trimester ↓ 2nd and 3rd trimesters	–	Evaluate correction bolus through the CHO/INS Add a “fake” quantity of CHO at meal bolus Increase max bolus if needed	Anticipate bolus (10–15′ in 1st trimester, 30′–45′ in 2nd–3rd)	Temp Target 150 mg/dL
Control-IQ	Basal rates CHO/INS ISF Body weight	112.5–120 Sleep mode in 24 h (no automatic correction boluses) Non-Adjustable	5 h Non-adjustable	↑ 1st trimester ↓ 2nd and 3rd trimesters (up to 400/TDD units/g)	Adaptable (up to 1620/TDD)	Does not accept reduction of bolus < 110 mg/dl Increase max bolus if needed Extended boluses [15 min-2 h]	Manual correction bolus e.g. 1–5 units no more than one every 2–3 h with noCHO/INS, especially post-dinner and bedtime Anticipate bolus 10–15′ in 1st trimester, 30′–45′ in 2nd–3rd	Exercise mode Target 140–160 mg/dL
DBLG1^	Glucose target Mean CHO consumed Body weight	100* mg/dl (from 100 to 130 mg/dl) Adjustable	Adaptive learning Automatically adjusted	Non-adjustable, bolus aggressiveness can be increased from the 2nd trimester	–	Manual correction bolus with no CHO/INS	Anticipate bolus (10–15′ in 1st trimester, 30′–45′ in 2nd–3rd)	Emergency carbohydrates** Sport mode, Zen mode Raised target for 1–8 h)
SmartAdjust^	Glucose target CHO/INS	110 mg/dl (from 110 to 150 mg/dl) Adjustable	2 h Adjustable	↑ 1st trimester ↓ 2nd and 3rd trimesters	Adaptable (affects TDD)	Evaluate correction bolus through the CHO/INS Turn “Reverse Correction” OFF	Anticipate bolus 10–15′ in 1st trimester, 30’–45′ in 2nd-3rd The lowest “Correct Above” threshold	Exercise mode Target 150 mg/dL

*There is no significant data on the recommended target in pregnancy, we recommend 100, being the most stringent target

**It recommends emergency carbohydrate if hypoglycaemia is expected in the next 15 min

^^^Update basal rate regularly to ensure adequate coverage in case of loop opening

^§^Update ISF regularly to ensure adequate coverage in case of loop opening

*CGM* continuous glucose monitoring, *AIT* active insulin time, CHO/INS *Carbohydrate-Insulin Ratio* *TDDI* total daily insulin dose, ↑ Increased, ↓ Decreased

## Device-specific strategies for optimizing fasting and nocturnal glycaemic control in pregnancy

Achieving optimal fasting and overnight glycaemic control is essential in the management of T1D during pregnancy, as elevated fasting glucose levels are associated with increased risk of adverse neonatal outcomes. While AID systems improve overall glucose control, current algorithms are not specifically designed to meet the stringent fasting targets recommended in pregnancy (70–95 mg/dL) [30]. The CamAPS FX system comes closest, allowing programmable targets between 80–95 mg/dL. All other systems operate above this range. As such, achieving optimal fasting control often requires device-specific strategies and manual adjustments tailored to the capabilities and limitations of each AID system. These adjustments may include pre-bedtime bolus corrections, fine-tuning of basal rates or active insulin time, and individualised glucose target settings where possible. The following strategies are based on clinical experience and supported by published evidence where available. Notably, if overnight hyperglycaemia is suspected to result from infusion set failure, correction insulin should be administered via subcutaneous injection (e.g., insulin pen) rather than through the pump, to ensure rapid and reliable absorption.

### CamAPS FX

Practice considerations:

- Set the overnight target to 80 mg/dL if tolerated, as this is the closest available setting to the pregnancy fasting goal (70–95 mg/dL).
- During the first trimester, consider a personal glucose target of 99 mg/dL
- Use the Boost function or administer manual correction boluses entries to address rising glucose levels overnight or delayed postprandial peaks.
- If fasting glucose remains elevated despite target settings, consider a manual bolus of 1–1.5 U before bed.
- In cases of persistent overnight hyperglycaemia or inconsistent loop performance, temporarily switch to SAP mode and apply a tailored manual basal profile.
- Adjust insulin total daily dose (TDDI) and body weight every 2–4 weeks to reflect evolving physiological insulin needs in pregnancy.
- Ensure stable Bluetooth connectivity overnight by keeping the smartphone within range (< 6 m) to maintain uninterrupted closed-loop function.

### Smart guard

Practice considerations:

- Use the 100 mg/dL target overnight, though this exceeds the recommended pregnancy fasting range (70–95 mg/dL).
- Consider entering fake carbohydrates at bedtime to trigger additional insulin delivery when correction boluses are insufficient for nocturnal hyperglycaemia.
- Educating patients on using the assisted bolus strategy to address elevated post-dinner or overnight glucose, especially when relying solely on auto-corrections may delay response [17, 102].
- Review and adjust the manual fallback settings as necessary every 3–4 weeks, to ensure they align with the insulin delivery patterns observed during automated mode (in case of loop interruption).
- Connectivity is integrated in the pump; SmartGuard does not rely on a smartphone or app for function

### Control-IQ

Practice considerations:

- Enable Sleep Mode (automatic correction boluses are disabled) overnight to tighten glycaemic control, although its fixed target range (~ 112.5–120 mg/dL) remains above the recommended fasting range in pregnancy (70–95 mg/dL).
- If fasting glucose remains elevated, consider a manual correction bolus or fake carbohydrate entry before bed, based on trend data.
- Adjusting AIT or ICR has no effect during closed-loop operation, so focus on optimising meal timing, carbohydrate estimation, and early post-meal bolusing to prevent overnight hyperglycaemia.
- To reduce the risk of rebound hyperglycaemia, minimise prolonged basal insulin suspension by reviewing basal timing and adjusting manual basal settings during suspension-prone hours.
- Ensure the smartphone remains within 6 m of the pump to maintain stable Bluetooth connectivity and uninterrupted Sleep Mode performance.

### DBLG1

Practice considerations:

- Use the 100 mg/dL target for overnight periods. The system adapts automatically but tends to operate above the recommended fasting range (70–95 mg/dL).
- Meal Assist or manual boluses/fake carbohydrate entries can be used to fine-tune insulin delivery, particularly in the case of late postprandial peaks or nocturnal excursions.
- Patients require structured training to understand when and how to override the loop with manual corrections, especially in situations where bolus aggressiveness is increased or the system is less responsive.
- Body weight, Initial Total Daily Insulin Dose (TDD), and Meal type declaration should be updated every 2–4 weeks to match evolving insulin resistance.
- Bolus aggressiveness and key algorithm settings are managed exclusively by the healthcare provider, which limits user autonomy and necessitates regular clinical review during pregnancy.

### Smart adjust

Practice considerations:

- Use the lowest available target (110 mg/dL) overnight, acknowledging that this may be insufficient to achieve fasting glucose goals of 70–95 mg/dL recommended in pregnancy.
- If fasting glucose remains elevated, consider administering a small manual correction bolus (1–1.5 U) or using fake carbohydrate entries before bed to prompt additional insulin delivery.
- In cases of persistent overnight hyperglycaemia, switching to manual mode overnight may provide greater control, with basal and correction settings adjusted accordingly.
- The system’s adaptive algorithm requires consistent use; ensure weight and insulin parameters are updated every 2–4 weeks to reflect evolving insulin needs during pregnancy.
- Ensure the smartphone remains within 6 m of the Pod during the night to maintain uninterrupted loop functionality by Bluetooth.

## Device-specific strategies for controlling postprandial hyperglycaemia in pregnancy

Effective control of postprandial hyperglycaemia during pregnancy requires a strong foundation in patient education and lifestyle management. Structured counselling should reinforce adherence to a healthy dietary pattern, emphasizing balanced meal composition, a preference for complex carbohydrates, and consumption of low glycaemic index foods [106]. Once lifestyle behaviours and carbohydrate estimation accuracy have been evaluated and optimized, device-specific strategies may be employed to enhance postprandial glucose control. These strategies should be individualised based on the patient's glycaemic response patterns, the composition of meals, and the individual’s confidence and training in using diabetes technology [107]. Ongoing support from a multidisciplinary team experienced in both pregnancy care and diabetes technology is critical for the safe and effective implementation of these strategies. Notably, while some approaches are routinely used in clinical practice, many remain based on expert consensus and clinical experience, with limited high-quality evidence from randomised controlled trials [85, 108]. Although no randomized clinical trials have formally evaluated a structured assisted-bolus protocol using SmartGuard^™^, experience reported in peer-reviewed literature—including case series in pregnancy and real‑world observations—supports the use of manual pre-meal or correction boluses to supplement AID in managing postprandial hyperglycaemia [102]. It is generally recommended to regularly review and update the maximum bolus setting in AID systems, ensuring it remains set above the typical bolus amount required for larger meals or correction doses. The following strategies are based on clinical expertise and published evidence, where available.

### CamAPS FX

Practice considerations:

- Boost function (increases insulin delivery by approximately 35% above the usual automated basal rate for up to 1 h) can be activated when upward trend arrows appear and glucose is > 140 mg/dL. For glucose > 180 mg/dL, consider adding a manual correction bolus instead of fake carbohydrates.
- Anticipate meal bolus timing: first trimester: 10–15 min pre-meal; Third trimester: up to 45 min pre-meal
- For post-breakfast hyperglycaemia, Boost can be pre-programmed the night before, set to start ~ 1 h before breakfast.
- The system does not allow disabling inverse correction (i.e., it reduces boluses when glucose is near or below target), but: define custom glucose targets by time segment (e.g., pre-meal), and set the minimum glucose value for bolus calculation as low as 50 mg/dL to minimize bolus reduction.
- Avoid using the "slow-absorption meal" function during pregnancy, as it may blunt necessary insulin response.
- Do not rely on “fake carbs” post-meal; use manual bolus for better control.

### SmartGuard

Practice considerations:

- Post-meal hyperglycaemia is often undercorrected due to the non-adjustable “safe meal bolus” function, which reduces insulin when hypoglycaemia is predicted.
- “Safe meal bolus” feature cannot be disabled; ICR should be aggressively adjusted, though this alone is often insufficient.
- “Fake carbohydrates” can be used at the time of meal to bypass bolus reduction: add 10–70% more carbohydrates than planned (adjusted by trimester and individual insulin sensitivity) to force a higher bolus (“assisted fake carbs”) [17, 102]. Postprandial correction with fake carbs can be avoided if the above strategy is used at the time of bolus.
- If late hypoglycaemia occurs, consider using the temporary target function to suspend auto-corrections for 1–2 h, starting 60–90 min after the meal [102].

### Control-IQ

Practice considerations:

- Postprandial corrections are limited by the algorithm's safety features and inability to override bolus reductions.
- For correction: use manual correction boluses, not more than every 2–3 h. Do not rely on fake carbs, especially during Sleep Mode.
- In cases of post-meal hyperglycaemia, apply a “super bolus” strategy [108]: Lower ICR to give more insulin upfront and temporarily reduce basal insulin for 2–3 h post-meal to prevent delayed hypoglycaemia (manual mode only).
- Inverse correction is optional for glucose < 110 mg/dL; it can be disabled to avoid under-dosing.

### DBLG1

Practice considerations:

- Bolus aggressiveness can be adjusted by the healthcare provider (from 50 to 200%) for specific meals.
- For recurrent postprandial hyperglycaemia, increase bolus aggressiveness for that meal.
- “Meal Assist” can be used to manage anticipated postprandial glucose excursions. When combined with accurate carbohydrate entry and appropriate meal type selection (e.g., light, usual, or large), it helps the algorithm deliver a more tailored insulin response.
- If needed, modify Total Daily Dose (TDD) as a surrogate for insulin sensitivity.
- Manual boluses are allowed, but require appropriate patient training.

### SmartAdjust

Practice considerations:

- Use manual bolus or fake carbs (e.g., 1–2 U equivalent) only after 2–3 h, to avoid insulin stacking.
- If post-meal hyperglycaemia is persistent, consider switching to manual mode, and adjusting ICR more aggressively.
- “Reverse correction” can be disabled to allow the bolus calculator to deliver the full calculated insulin dose for carbohydrates, without reducing it when pre-meal glucose levels are at or below target.

## Device-specific strategies for managing hypoglycemia in pregnancy

Although AID systems have significantly reduced the incidence and severity of hypoglycaemia in type 1 diabetes during pregnancy, episodes still occur and must be carefully managed [109]. It is important to highlight that, in AID systems, treating hypoglycemia with reduced amounts of carbohydrates (e.g., 4–7 g) compared to traditional protocols (e.g., 15 g) may be more effective in restoring normoglycemia without causing rebound hyperglycemia especially because the sensor glucose lower limit is considered 63 mg/dL, not 70 mg/dL [30]. Only certain systems, such as DBLG1 and CamAPS FX, allow manual entry of carbohydrates consumed for hypoglycemia correction, enabling the algorithm to better modulate insulin resumption. Other systems, such as Control IQ, SmartGuard and Smart Adjust, do not offer this functionality, leaving the system unaware of carbohydrate intake and increasing the risk of overcorrection and rebound hyperglycemia.The following strategies are based on clinical expertise and published evidence, where available.

### CamAPS FX

Practice considerations:

- Suggested hypoglycaemia treatment doses: 4 g of carbohydrate for a single downward trend arrow and 8 g of carbohydrate for two downward trend arrows.
- The system includes a “Hypoglycaemia Treatment” feature, then when activated, it displays the intake as a meal on the detailed graph. Importantly, the algorithm does not deliver insulin to cover this marked treatment, reducing the risk of rebound hypoglycaemia.
- The Ease-Off feature temporarily reduces insulin delivery and raises the system’s glucose target [110]. CamAPS allows for manual boluses to be withheld, and targets can be increased temporarily if persistent hypoglycaemia is anticipated (e.g., during activity).

### SmartGuard

Practice considerations:

- The system does not allow users to communicate hypoglycaemia treatment to the algorithm.
- Emergency carbs (typically 7–10 g) may suffice due to proactive basal suspension.
- A rapid glucose rise following carb intake may trigger auto-correction boluses, potentially leading to recurrent hypoglycaemia.
- The user can select a temporary target (TT) of 150 mg/dL. This disables the administration of correction boluses. It does so for as long as the TT is activated [111].

### Control-IQ

Practice considerations:

- Control-IQ does not recognize hypoglycaemia treatments and cannot suspend boluses already delivered.
- Enabling Sleep Mode (which disables automatic corrections and tightens the target range) can help prevent post-treatment overcorrection.
- Activity Mode raises the system’s glucose target (to ~ 150 mg/dL) and reduces insulin delivery.
- If the patient experiences hypoglycaemia despite activating the exercise mode, a good option is to create a less aggressive personalised profile by decreasing the basal rate by 30–50%, and increasing the CF and ICR by 40–50% [112].

### DBLG1

Practice considerations:

- DBLG1 includes predictive hypoglycaemia management:the system automatically suggests an emergency carbohydrate dose when hypoglycaemia is predicted within 15 min. Carb amount is individualized based on patient settings and insulin history.
- Carb intake recommendations are visible to the user, and the algorithm accounts for this in future insulin delivery.
- The Physical Activity mode can be used. In this mode, the glucose target and hypoglycaemia threshold are increased by 70 mg/dL, which reduces the aggressiveness of insulin delivery.
- Another feature of the DBLG1 system is the ‘ZEN’ mode, which increases the glucose target by an increment that is between 10 and 40 mg/dL for a period of 1–8 h mode (not a pregnancy-specific mode) [113].

### SmartAdjust

Practice considerations:

- The algorithm suspends insulin automatically in anticipation of lows. No feature exists to “announce” carb intake for hypoglycaemia treatment.
- Raise the GT level by 10 mg/dl. For hypoglycemic episodes following user-initiated correction boluses, consider raising the “correct above” threshold or increasing the
- Enabling the Activity feature sets the Target Glucose to 150 mg/dL and reduces automated insulin delivery.

### DIY (do it yourself)

#### Rationale for use

DIY artificial pancreas systems (DIY APS) are unregulated hybrid closed-loop insulin delivery systems developed by members of the T1D community using open-source software (e.g., Loop, AndroidAPS, OpenAPS). These systems integrate continuous glucose monitoring (CGM) and insulin pump technology to algorithmically adjust insulin delivery in real time, aiming to maintain glucose levels within a predefined range. Despite lacking regulatory approval, DIY APS have gained popularity due to their high degree of customisation, flexibility in glycaemic targets, and broader bolus delivery options than commercially available AID systems. The appeal is especially strong among experienced users and parents of children with diabetes, many of whom report improved glycaemic control, reduced glucose variability, better quality of life, and improved sleep [114, 115].

#### Scientific evidence and recommendations

The use of DIY APS during pregnancy remains an area of growing interest, though the current evidence base is still limited. Initial reports focused on individual cases [116, 117], who described the use of the Loop algorithm in pregnant women with T1D. In both cases, maternal–fetal outcomes were favorable, with time in range (TIR) exceeding 70% by the third trimester and time below range (TBR) maintained below 5%, aligning with glycaemic targets recommended in pregnancy. More recently, additional publications have expanded our understanding of DIY APS use in this context [118, 119] described multiple pregnancies managed with Loop. The authors reported TIRs greater than 75% during the second and third trimesters, with no instances of severe hypoglycaemia or diabetic ketoacidosis (DKA). Neonatal outcomes were also reassuring, with all infants appropriate for gestational age and no admissions to neonatal intensive care. Most recently, Sobhani et al. [4] published a more comprehensive retrospective series examining eight pregnancies in women using the Loop algorithm. The mean TIR during the third trimester was 78%, with no episodes of severe hypoglycaemia or DKA reported. Neonatal outcomes were favorable across the cohort, with birth weights within the normal range and no major complications. The authors highlighted the proactive role of patients in configuring and adjusting system parameters as a key factor in achieving optimal results. The available evidence is limited to small case reports and retrospective series, lacks randomized controlled trials, and reflects highly selected, motivated patients using non-regulated technologies, which restricts the generalizability of these findings. Due to the scarcity of robust data, any clinical implications must be considered preliminary and interpreted with great caution rather than framed as practical recommendations.There are currently no data on the use of DIY APS in pregnant women with type 2 diabetes.

#### Suggestions for use in clinical practice

While DIY artificial pancreas systems (APS) are not approved for use in pregnancy, emerging evidence and user experiences offer insights that can guide safe and effective use in motivated individuals. Some advice on the use of DIY systems in pregnancy, are based on published data [114–118] and anecdotal experience.

1. *Glycaemic targets*. Aim for glucose targets consistent with pregnancy guidelines: Fasting glucose: 85–90 mg/dL; Postprandial glucose: 100–110 mg/dL. Configure system settings to reflect these targets as closely as possible.
2. *Bolus strategies for pregnancy*. Due to delayed gastric emptying in pregnancy, particularly in the second and third trimesters, use extended bolus strategies:
  Breakfast: Use the LOLLIPOP bolus (carbohydrate absorption set to 2 h).
  Low-fat meals (lunch/dinner): Use Taco bolus [3 hours duration], or Pizza bolus (4 hours duration).
  Encourage pre-bolus timing of 15–30 minutes before meals to reduce postprandial spikes.
3. *System settings adjustments*. Low glucose suspend threshold: Lower this to 65 mg/dL during pregnancy to avoid severe hypoglycaemia. Active insulin time (AIT): Prefer a 6-h AIT to reduce insulin stacking. If switching from a shorter AIT (e.g., 3 h), consider adjusting the insulin sensitivity factor (ISF) to avoid under-correction. Use a less aggressive correction factor if needed, especially in the third trimester, to prevent overcorrection [93].
4. *Device transitions*. When transitioning from other AID systems to a DIY APS, assess how prior settings (e.g., AIT, ISF, basal patterns) may need recalibration. Update conventional pump settings regularly to ensure safety in the event of system disconnection or fallback to manual mode.
5. *System downtime awareness*. Reinforce to patients that any period outside of closed-loop mode (e.g., CGM signal loss or pump disconnection) can impact glycaemic control. Encourage frequent system checks and prompt troubleshooting.
6. *Education and support*. Offer individualized counselling that includes a transparent discussion of the off-label nature and potential risks of DIY APS in pregnancy. Limitations due to unregulated software, out-of-warranty hardware, and lack of standardized healthcare provider training. Collaborate closely with patients to monitor and adjust settings throughout pregnancy.

## Practical guidance and areas of uncertainty in the use of automated insulin delivery (AID) systems during pregnancy

The use of AID systems during pregnancy offers the potential for improved glycaemic outcomes, reduced hypoglycaemia risk, and enhanced quality of life. However, clinical practice remains challenged by significant variability in regulatory approvals, algorithm design, and evidence supporting the safety and efficacy of these systems in pregnant individuals with T1D. Based on the evidence and expert consensus presented, the following key messages emerge:

Key practical messages:

1. CamAPS FX and SmartGuard (MiniMed^™^ 780G) are currently the only CE-marked AID systems approved for use in pregnancy, with CamAPS FX also carrying FDA approval and offering the most adjustable glucose targets aligned with pregnancy-specific fasting recommendations (80–95 mg/dL); while SmartGuard (MiniMed^™^ 780G) permits a minimum target of 100 mg/dL under its most aggressive settings.
2. All other commercial AID systems operate with targets above the recommended pregnancy range and require individualized optimization strategies.
3. Postprandial glycaemic control remains a major challenge, particularly due to conservative bolus algorithms, non-modifiable features (e.g., "safe meal bolus"), and delayed insulin absorption in later trimesters. Strategies such as bolus timing adjustments, aggressive ICR modification, and selective use of fake carbs are essential.
4. Hypoglycaemia management must be adapted, using smaller carbohydrate doses (4–10 g) and avoiding overcorrection, particularly in systems unable to recognize treatment intake. Predictive features and carb tagging (available in DBLG1 and CamAPS FX) can improve safety.
5. Structured education and multidisciplinary care are critical. All users should be trained to revert to manual or open-loop modes in the event of suboptimal control or device malfunction, and closely monitored by a team experienced in pregnancy and diabetes technology.
6. Use in pregnancy remains off-label for most systems (except CamAPS FX and MiniMed^™^ 780G in Europe). Informed consent, individualized risk–benefit assessment, and ongoing supervision are mandatory.
7. Night-time glycaemic control is generally more responsive to automation than daytime, but fasting targets often remain unmet. Tailored overnight strategies are required to address this gap.

Areas of ongoing uncertainty and research need:

- Lack of pregnancy-specific algorithms that account for physiological insulin resistance and variability in gastric emptying.
- Inconsistent data on maternal–fetal outcomes and the long-term safety of AID use during pregnancy.
- Insufficient integration of pregnancy-specific metrics (e.g., psTIR, psTBR) in routine reports across all platforms.
- Psychosocial impacts of AID use in pregnancy remain underexplored and variable across user populations.
- DIY APS systems (e.g., Loop, AndroidAPS) remain unregulated and are not formally recommended for pregnancy.

In conclusion, while AID systems hold promise for improving diabetes management in pregnancy, their use must be personalized, closely monitored, and grounded in a strong foundation of education and expert care. Future research and algorithm development should prioritize pregnancy-specific adaptations to maximize safety and efficacy in this uniquely complex population.

## The use of technology in the peripartum period

### Blood glucose monitoring

#### Rationale for use

The main goal of labor management in pregnant women with diabetes is to prevent maternal hyperglycemia, avoid hypoglycemia, acidosis, and fetal hypoxia. The desirable blood glucose level during labor and delivery should be between 70 and 126 mg/dL [9]. However, recent evidence suggests that a less stringent blood glucose range (90–140 mg/dL) may also be considered to reduce the risk of maternal hypoglycemia and the use of insulin infusion therapy [8]. Rapid changes in glucose and insulin requirements during labor and delivery necessitate frequent blood glucose monitoring; it should be performed hourly in active labor, if blood glucose levels remain stable. Optimal blood glucose levels should also be maintained during delivery, whether vaginal or by cesarean section. Frequent glucose dipstick testing can be stressful for women in labor and can be a burden for healthcare providers in the labor and delivery room. CGM systems offer the ability to continuously monitor glucose levels, even when the user is away. The use of CGM during pregnancy is growing, with evidence suggesting that CGM can help achieve optimal pre- and post-prandial glucose values in pregnant women with T1D, improving maternal and neonatal outcomes. Flash scanning continuous glucose monitoring (FGM) systems require scanning the sensor to provide a glucose reading. Although this requires moving the reader closer to the sensor, it provides a glucose reading more quickly and easily than SMBG.

#### Scientific evidence and recommendations

Few studies have evaluated the accuracy of FGM and CGM during labour and delivery, but overall their use appears to be feasible, accurate and clinically useful [120, 121]. A retrospective analysis comparing CGM (Dexcom G6) and point-of-care SMBG in women with T1D found good overall accuracy for CGM (Dexcom G6) during labour and delivery, with a median absolute relative difference (ARD) of 6.1%, but significantly lower accuracy for hypoglycaemia (median ARD 44.3% for glucose < 70 mg/dL) [104]. Glucose/insulin infusion rate decisions during labour and delivery were 75% more consistent with CGM (Dexcom G6) than with SMBG [103]. Similarly, real-world data show good accuracy of the CGM system (FreeStyle Libre 1 and 2) during caesarean section in women with insulin-treated diabetes (T1D, T2D and GDM), with MARD of 9.28% compared to capillary glucose, which improved to 8.82% when excluding glucose values ≤ 70 mg/dL [122]. In addition, 100% of interstitial glucose values fell within the "clinically acceptable" Clarke Error Grid zones A and B [122]. Agreement of values to guide decision making according to the glucose/insulin infusion protocol used was achieved in 68% of cases overall, with complete agreement for values between 70 and 110 mg/dL [122]. Finally, there is an increasing number of clinical cases [123] of the use of automated insulin-delivery systems (AIDs) during pregnancy, together with evidence from clinical trials [92, 102] that most women have chosen to maintain AID during labour and postpartum. In AID systems, the subcutaneous insulin infusion delivered by the pump is automatically adjusted based on the interstitial glucose value read by the CGM. The results observed are encouraging and support the possibility of using CGM during labour and delivery. The current (2018) Italian standards of care for AMD-SID [9] recommend very low blood glucose levels (70–126 mg/dL) during labour and delivery and consider frequent capillary blood glucose monitoring necessary to achieve these levels. Similarly, the National Institute for Health and Care Excellence guidelines [11] recommend hourly capillary blood glucose monitoring during labour and delivery in women with diabetes and maintaining it between 72 mg/dL and 126 mg/dL. The Joint British Diabetes Societies (JBDS) guidelines (2023) [55] recommend that blood glucose should be monitored hourly during labour or from the morning of elective caesarean section, or every half hour if general anaesthesia is used, with the option of capillary dipstick or FGM or CGM monitoring to maintain glucose levels (capillary, flash or CGM) within the recommended target range (72–126 mg/dL or the less restrictive range of 90–144 mg/dL) during labour. However, they recommend that capillary glucose should always be used to adjust the glucose/insulin infusion.

#### Suggestions for use in clinical practice

The use of CGM during labour and delivery can allow women to be closely monitored more easily than with capillary glucose monitoring. In addition, CGM systems provide more information than capillary glucose monitoring: the trend arrow can predict glucose levels, allowing corrective action to be taken in advance, if necessary. In addition, all modern CGMs have alarm systems to detect hypoglycaemia and hyperglycaemia that may occur outside of scheduled glucose testing times. When using a CGM or FGM monitoring system in the peripartum period, the condition of the sensor and the charge of the device used to read the sensor should be checked. Replace the sensor if it is about to expire, allowing for a new sensor activation time of between 30 and 120 min, depending on the system, during which no glucose values will be recorded. CGM or FGM monitoring systems are useful to manage insulin therapy in the peripartum period. However, confirmation with a capillary glucose stick is recommended if glucose levels are < 70 mg/dL and before changing the infusion rate. In the case of a caesarean section, women should move the sensor to the arm at least the day before surgery so as not to interfere with the surgical field. In the postpartum period, the FGM or CGM can be used, with the glucose confirmation stick used only to check for hypoglycaemia or to change the infusion rate, if still in progress. For women who are breastfeeding, it is recommended to check glucose levels before breastfeeding and to consume 10–15 g of carbohydrates if glucose levels are below 110 mg/dL (a glass of milk can be a good snack, also because it promotes hydration).

### Continuous subcutaneous insulin injections (CSII)

#### Rationale for use

Planning insulin management during labour and delivery is an integral part of the care of pregnant women with diabetes. The aim is to avoid maternal hypoglycaemia while preventing significant hyperglycaemia, which can increase the risk of neonatal hypoglycaemia. CSII management should be adaptable to the different needs and stages of labour and delivery, including possible dietary restrictions and the need for surgical intervention. During labour and delivery, intravenous insulin therapy is the most commonly used treatment option for glycaemic control, but there is growing evidence that CSII therapy can be continued throughout labour and delivery.

#### Scientific evidence and recommendations

A real-world Italian study documented that CSII can be used effectively and safely during labour in well-trained and motivated women with T1D [124]. The efficacy of CSII was demonstrated by maintaining maternal peripartum glucose levels within target range throughout the period in over 80% of the 65 women included in the study; comparable and stable values were also found in the group of women who underwent caesarean section [124]. More recently, another retrospective cohort study of intrapartum glycaemic control in pregnant women with T1D confirmed that the use of CSII during labour and delivery appears to be a comparable option to intravenous insulin infusion in terms of perinatal outcomes [125]. Convincing data are also emerging from a recent RCT [126] in women with T1D, which showed no differences in the primary outcome of neonatal hypoglycaemia between women who continued CSII therapy during labour and those who used intravenous insulin infusion, suggesting that women should be given the option of choosing either strategy for managing therapy during labour, in line with what has previously been communicated to the diabetologist. The current (2018) Italian standards of care for AMD-SID [9] indicate that the use of CSII during labour and delivery may be useful to maintain good metabolic compensation, if the team attending the patient during delivery is trained in CSII management. Good glycaemic control can be achieved with a basal rate reduced by 50% from the start of the active phase of vaginal labour or from the start of anaesthesia for caesarean section. Similarly, the 2018 Diabetes Canada Clinical Practice Guidelines [54] state that options for peripartum glycaemic control include continuing CSII therapy for women who wish to do so and recommend that basal insulin administered via CSII be reduced by at least 50% immediately after delivery to avoid hypoglycaemia. The Joint British Diabetes Societies (JBDS) 2023 guidelines [55] state that women on CSII therapy may choose to continue CSII therapy during the intrapartum and postpartum periods, but recommend pump removal if the woman is unable to manage the situation, if glycaemic control becomes unstable or worsens, or if ketones occur.

#### Suggestions for use in clinical practice

Maintaining CSII in the intrapartum period appears to be a reasonable option in women with T1D, as no difference in maternal or neonatal outcomes was observed compared with intravenous insulin infusion. This glycaemic control strategy may be valid in women who are highly motivated to continue CSII during labour and delivery and who have received prior training from the multidisciplinary team. However, continuation of CSII therapy will only be possible if a multidisciplinary team with diabetologists experienced in the management of diabetes in pregnancy and/or a trained nurse capable of managing intrapartum CSII is available. In addition, women should be informed before the intrapartum period about the expected glycaemic changes during latent labour, active labour and immediately after delivery. However, in the event of deterioration in glycaemic control and in conditions where the patient's cognitive status is impaired (pain or general anaesthesia), it is advisable to suspend the device and switch to patient-independent management. The diabetologist must explain to the patient that near the delivery the pump cannula must be positioned well away from the potential surgical field. The patient must also make sure that she has enough battery and insulin for labour and delivery.

At the last antenatal visit, the diabetologist should set 3 different basal infusion rate profiles to be activated during the different phases of labour and delivery, based on blood glucose levels [124]:

- Profile A: basal rate adopted up to that moment
- Profile B: basal rate halved compared to Profile A (50% of Profile A)
- Profile C: safety, with basal rate of 0.1–0.2 IU/h. In the case of using PLGS systems, this profile will not be necessary since the algorithm suspends the basal infusion 30’ before any hypoglycemia.

During induction and the latent phase of labour, if blood glucose levels are between 70 and 140 mg/dL, you can continue with profile A, possibly giving small corrective boluses (0.5–2 units) if blood glucose levels rise to 180 mg/dL. If blood glucose remains above 180 mg/dL, consider switching to intravenous insulin infusion (according to specific hospital protocols). For blood glucose levels between 50 and 70 mg/dL, use Profile B (50% of Profile A). For blood glucose levels lower than < 50 mg/dL, switch to Profile C (basal rate of 0.1–0.2 IU/h) combined with 10% IV glucose solution. When using PLGS systems, this profile is not necessary as the algorithm stops the basal infusion 30 min before hypoglycaemia occurs. During the active phase of labour or before anaesthesia in the case of caesarean section, if blood glucose levels are between 140 and 180 mg/dL, you can continue with profile A, combining 5% glucose solution at 80 ml/h, possibly with small corrective boluses (0.5–2 units, depending on the sensitivity factor). If blood glucose remains above 180 mg/dl, consider switching to intravenous insulin infusion (according to specific hospital protocols). For blood glucose levels between 70 and 140 mg/dL, use profile B (50% of profile A), combining 5% glucose solution at 80 ml/h. For blood glucose levels between 60 and 70 mg/dL, use profile C (basal rate of 0.1–0.2 IU/h), combining 5% glucose solution at 80 ml/h. For blood glucose levels < 60 mg/dL, switch to profile C (basal rate of 0.1–0.2 IU/h), combining 10% glucose solution at 100 ml/h. If PLGS systems are used, this profile is not necessary as the algorithm stops the basal infusion 30' before hypoglycaemia occurs. In the postpartum period, until oral feeding is resumed, for blood glucose levels between 70 and 180 mg/dL, profile B can be used, combining 5% glucose solution at 80 ml/h. If blood glucose is between 180 and 250 mg/dL, use profile B, combining 5% glucose solution at 80 ml/h, possibly with small corrective boluses (0.5–2 units, depending on the sensitivity factor). If blood glucose remains above 250 mg/dL, consider switching to intravenous insulin infusion (according to specific hospital protocols). For blood glucose levels < 70 mg/dL, switch to profile C (basal rate of 0.1–0.2 IU/h), combining 10% glucose solution at 100 ml/h. If PLGS systems are used, this profile is not necessary as the algorithm will stop the basal infusion 30 min before hypoglycaemia occurs. As soon as possible after delivery, the pump settings will be reassessed by the healthcare team and in most cases it will be recommended that the pump settings for insulin/carbohydrate ratio and sensitivity factor are restored to pre-pregnancy levels. A further 20% reduction in basal infusion is also appropriate when breastfeeding. When women stop breastfeeding/expressing, it may be necessary to adjust the pump settings, particularly the basal rate.

### Automated insulin delivery system (AID)

#### Rationale for use

The physiological stress and rapid changes in insulin requirements during labour and delivery in women with pre-pregnancy diabetes, and the care provided by obstetric staff with limited expertise in diabetes technology, represent a challenge to optimising glycaemic control during this delicate period of pregnancy. The automated nature of closed-loop systems and their ability to adapt to real-time glucose levels may make them useful tools during labour, delivery and the immediate postpartum period in women with T1D who have used them during pregnancy.

#### Scientific evidence and recommendations

In a study using the Cambridge Artificial Pancreas System (CamAps FX) initially at night and then throughout the day, the majority of women using AIDs [14 out of 16] chose to keep the system until delivery, with a TIR of 87% during labour and delivery. The system independently reduced total daily insulin by approximately 50% immediately after delivery [93]. An analysis of two AID studies evaluated data from women with T1D who used the CamAPS algorithm closed-loop system during pregnancy and maintained it through labour and delivery, showing good results [93]. Women spent > 80% of the time in target range and the system was effective in maintaining tight glycaemic control during both vaginal delivery and caesarean section under regional or general anaesthesia [93]. The system adapted quickly to rapidly changing insulin needs after delivery, allowing women to spend over 80% of their time in target range in the immediate postpartum period [85]. The system has also been used with diathermy without complications [93]. In the CRISTAL trial [127], use of the MiniMed 780G system was associated with more TIR in women who continued to use it during delivery (71.5 ± 17.7% vs. 63.1 ± 17.0%, P = 0.030), and numerically lower TAR (27.3 ± 17.4% vs. 35.3 ± 17.5%, p = 0.054), without increases inTBR (1.1 ± 2.4% vs. 1.5 ± 2.3%, P = 0.146), compared to standard therapy, regardless of whether the delivery was vaginal or caesarean [127]. In the immediate postpartum period, women on AID who increased their insulin/carbohydrate ratio by an average of 67% maintained good glycaemic control, similar to women on standard insulin therapy (86.8 ± 6.7% vs. 83.8 ± 8.1%, p = 0.124) [127]. These findings suggest that AID is effective in maintaining tight glycemic control intrapartum and early postpartum and can be safely continued during periods of rapidly changing insulin requirements.

There is increasing evidence from real-world studies of women who have used AID during pregnancy and successfully maintained it until delivery [99, 102]. Retrospective Italian data on a small cohort of very fit women with T1D using the MiniMed 780G document optimal glycaemic control during labour and delivery (median ps-TIR 92.5%) and in the first 30 days postpartum, without episodes of severe hypoglycaemia. The authors chose to use the 120 target as a precautionary measure in view of the abrupt drop in insulin requirement after delivery, also considering the lack of information on the timing of algorithm adaptation to this change [102]. In the cohort described by Wang, all women successfully used the AID with Control-IQ technology during labour, after adjustment of insulin profiles before and after delivery, in the absence of hypoglycaemic episodes during labour, delivery or the first day after delivery [99]. The postpartum period is another delicate period for women with T1D, requiring considerable effort to maintain good glycaemic control. Infant care, breastfeeding, changes in daily routines, sleep deprivation, changes in insulin sensitivity and fear of hypoglycaemia during breastfeeding complicate insulin therapy management. In this context, the use of AIDs could represent a simplification in diabetes management, but data to support their use in the postpartum period are still limited. In a prespecified 6 months postpartum extension phase of AiDAPT study with CamAps FX system, mean time with glucose levels within the target range was higher in the AID group compared with the standard care group (72 ± 12% vs. 54 ± 17%), with an adjusted treatment difference of 15% [128]. Glycaemic improvements were met by a marked reduction in maternal hyperglycaemia, especially evident overnight, and the improvements were not accompanied by an increase in hypoglycaemia (2.4% vs. 2.6%) [128]. Post hoc analysis of maternal glycaemia over the first 2 weeks postpartum starting from the day of delivery demonstrated the immediate beneficial effect of AID versus standard insulin therapy with CGM use (AID TIR 80% vs. standard care TIR 67%) [128]. Differences in glycaemia were also apparent at 4 weeks postpartum and in each subsequent 4-week period following delivery, with consistently higher TIR for the AID group, suggesting consistency of the treatment effect during postpartum period and supporting continued use of AID rather than standard insulin therapy for women with T1D once they have given birth [128].

In the CLIMB study [129], postpartum women with T1D were randomised to receive insulin using the MiniMed 670G/770G system in automatic mode or sensor-assisted pump therapy for the first 12 weeks postpartum and then in automatic mode up to 24 weeks postpartum. In this study, no significant differences in TIR were observed, but there was a reduction in the time spent in hypoglycaemia in women using postpartum AID, which was positively perceived by women using the AID system [130]. A secondary analysis of the study showed that during the breastfeeding period, there was a slight reduction in maternal glucose levels recorded by CGM systems in the 3 h following overnight breastfeeding, but this did not reach the hypoglycaemic threshold; this trend was more attenuated in women using AID [131]. The current (2018) Italian AMD-SID standards of care [9] indicate that the use of AIDs during labour and delivery may be helpful in maintaining good metabolic compensation. The 2023 Joint British Diabetes Societies (JBDS) guidelines [55] state that women on AID therapy may choose to continue AID therapy during the intrapartum and postpartum period; these women should have individualised care plans.

#### Suggestions for use in clinical practice

The use of AID systems during labour and delivery is increasing, may help to maintain tight glycemic control and offers advantages for both women and health care professionals. It is currently possible to provide some suggestions drawn up by physicians more experienced in the use of AIDs on the basis of the indications provided by the clinical studies available to date (Table 10). For all AID systems, it is suggested to set the most restrictive glycaemic target allowed by the specific system during labour and delivery. It is also recommended to define, already during the last pregnancy visit, safety basal profiles to be used in case the target is not reached, making it necessary to switch from automatic to manual mode. In this case, please refer to the indications suggested for the use of CSII during labour and delivery. In the postpartum period, given the reduced need for insulin, it is advisable to make some adjustments as soon as possible to reduce the risk of hypoglycaemia (Table 10). Women should be advised to switch to recommended postpartum settings immediately before caesarean section or as soon as the placenta is delivered.

**Table 10:** Postpartum diabetes management: automated insulin delivery system settings

	CamAPS FX [128]	Smart Guard [127]	Control I-Q [99]
Glucose target	> 108 mg/dL	100 or 120 mg/dL	–
Active insulin time	Adapt as needed	2 h	–
CHO/insulin	Bring back to values close to those pre-pregnancy	Bring back to values close to those pre-pregnancy	Bring back to values close to those pre-pregnancy
Insulin sensitivity	–	–	Adapt as needed
Body weight	Update	–	Update
Basal rate	50% of the basal rate at the end of pregnancy (only for manual mode)	50% of the basal rate at the end of pregnancy (only for manual mode)	2/3 of the pre-pregnancy basal rate (or 50% of the basal rate at the end of pregnancy)
Hypoglycemic trend	*Ease-off* mode (reduce basal rate and stop < 126 mg/dL)	Temporary target 150 mg/dL	*Exercise* mode

For the CamAPS system, the last programmed settings during pregnancy can be maintained during labour and delivery, using the "boost" and "ease-off" modes to continue to modulate basal insulin delivery according to current needs. In the event of a caesarean section, it is recommended that the system settings are updated to post-partum values prior to surgery. Again, the boost and ease-off modes can be used to continue to modulate basal insulin delivery based on current needs. Recommended initial postpartum settings included a personal glucose target of 108 mg/dL and insulin to carbohydrate ratios of between 1:12 g and 1:15 g, depending on infant feeding status. Following delivery, women should titrate their own personal glucose targets, insulin to carbohydrate ratios, and pre-meal insulin boluses, aiming for CGM TIR targets and should be encouraged to use the boost and ease-off features for at least 2–4 h at a time if they felt that other setting changes were not fast enough to counter higher or lower glucose levels [128].

For the MiniMed 780G system, the same settings of pregnancy period can be maintained during labour. Most women could use MiniMed 780G system postpartum with a glucose target of 100 mg/dL and active insulin time of 2 h, but in case of hypoglycemic trend the target should be sometimes increased to 110 or 120 mg/dL. Additionally, it is recommended to increase the insulin-to-carbohydrate ratios at least 50–65% during the end of the dilation phase and delivery, as a rapid decrease in insulin requirements is expected with delivery of the placenta [127].

For the Control-IQ technology the system should be switched to regular Control-IQ (not Sleep mode) at the start of labor or fasting for cesarean section. A postpartum profile with 2/3 pre-pregnancy basal rates (or 50% end of end of pregnancy basal rate) should be programmed during the last pregnancy visit. Moreover, it is suggested that there are 10% weaker correction factors and 10 to 20% reduction of insulin to carbohydrate ratios than pre-pregnancy. Women should be advised to switch to a post-partum profile just before caesarean birth or at the start of pushing. During labour and delivery sleep mode can be kept active. During labour, delivery, early post-partum and breastfeeding, exercise mode can be set in case of hypoglycemic trend [99]. For DBLG1 and SmartAdjustto date, there are no published studies or clinical guidelines evaluating the safety, efficacy, or outcomes associated with the use of DBLG1 or its SmartAdjust feature during the peripartum or postpartum periods.

## Conclusion

The use of technology in diabetes management during pregnancy is evolving rapidly, offering significant potential to improve glycaemic outcomes, reduce maternal and fetal risks, and enhance quality of life. However, the adoption of technologies such as continuous glucose monitoring (CGM), continuous subcutaneous insulin infusion (CSII), sensor-augmented pumps (SAP), and automated insulin delivery (AID) systems must be grounded in robust clinical understanding and adapted to the unique demands of pregnancy. Based on current international guidelines, emerging evidence, and expert experience, the following key practical messages can guide clinical care:

- *CGM is now a cornerstone of care* in type 1 diabetes during pregnancy and has a growing role in type 2 diabetes and gestational diabetes mellitus (GDM). Real-time CGM (rtCGM) offers greater benefit than intermittent scanning (isCGM), particularly for insulin-treated patients.
- *Pregnancy-specific glycaemic targets remain difficult to achieve*, particularly fasting and postprandial goals. Technology can support improved pregnancy-specific time in range (psTIR), but no system fully accounts for the physiological insulin resistance or delayed absorption unique to pregnancy.
- *CamAPS Fx is the only algorithm that currently holds FDA approval, while both CamAPS Fx and MiniMed 780G (SmartGuard algorithm) hold a CE marking for use in pregnancy*. Other AID systems and DIY APS platforms are used off-label and require structured education, informed consent, and close supervision.
- *DIY systems (e.g., Loop, AndroidAPS)* are increasingly used despite the absence of regulatory approval. Clinicians should provide patient-centred, nonjudgmental support to ensure safe use in those who opt for these systems.
- *Multidisciplinary team care* is critical. The safe use of advanced diabetes technology during pregnancy requires coordination among diabetologists, obstetricians, diabetes educators, and dietitians familiar with both pregnancy physiology and device management.
- *Technology use requires motivated, well-educated patients*. Engagement should ideally begin in the preconception phase, especially for those with pre-existing diabetes.

While real-world adoption of diabetes technology in pregnancy is increasing, several areas remain underdeveloped:

- Most AID systems are not designed specifically for pregnancy and lack algorithm adaptations for gestational physiology.
- High-quality, prospective data on maternal–fetal outcomes remain limited.
- Evidence for AID use in GDM and T2D is sparse, and research on psychosocial impacts is inconsistent.
- DIY APS systems are supported largely by observational evidence and real-world case series rather than randomized clinical trials

The integration of diabetes technology into pregnancy care represents a pivotal advancement in the management of diabetes during gestation. Despite important limitations—including the lack of pregnancy-specific algorithms, inconsistent regulatory approvals, and limited high-quality outcome data—current evidence and clinical experience support the thoughtful application of technologies such as continuous glucose monitoring (CGM), sensor-augmented pumps (SAP), and automated insulin delivery (AID) systems as adjuncts to improve glycaemic control and mitigate maternal–fetal risk. The Italian Diabetes Associations, AMD and SID, and the Interassociative Diabetes and Pregnancy Study Group, recognise the need for the cautious yet proactive implementation of these tools, adapted to individual clinical circumstances, based on the best available evidence. In this context, diabetes technology should be offered as part of an integrated, multidisciplinary care model accompanied by structured patient education, ongoing professional training and equitable access across regions and populations. Continued clinical innovation, robust pregnancy-specific trials and adaptive algorithms better aligned with gestational metabolic demands are strongly encouraged.

## Abbreviations

AID: Automated insulin delivery
AUC: Area under the curve
BMI: Body mass index
CGM: Glucose continuous monitoring
rt: Realtime
is: Intermittent scanned
CSII: Continuous subcutaneous insulin infusion
DKA: Diabetic ketoacidosis
FLS: FreeStyle libre
GDM: Gestational diabetes mellitus
HbA1c: Glycated hemoglobin
LGS: Low glucose suspend
MBG: Mean blood glucose
MDI: Multi-daily injection
PLGS: Predictive low suspending glucose pump
RCT: Randomized clinical trial
SAP: Sensor-augmented pump
SMBG: Self-monitoring blood glucose
T1D: Type 1 diabetes
T2D: Type 2 diabetes
TBP: Temporary basal profile
TDD: Total daily dose
TIR: Time in Range
DIY APS: Do it yourself artificial pancreas systems
